# Chemical Composition of Juices Made from Cultivars and Breeding Selections of European Pear (*Pyrus communis* L.)

**DOI:** 10.1021/acs.jafc.2c00071

**Published:** 2022-04-15

**Authors:** Wenjia He, Oskar Laaksonen, Ye Tian, Tuuli Haikonen, Baoru Yang

**Affiliations:** † Food Chemistry and Food Development, Department of Life Technologies, University of Turku, FI-20014 Turku, Finland; ‡ Production systems/Horticulture Technologies, Natural Resources Institute Finland (Luke), Toivonlinnantie 518, Piikkiö FI-21500, Finland

**Keywords:** cultivars, phenolic profile, perry pears, dessert pears, UHPLC-DAD-ESI-QTOF-MS

## Abstract

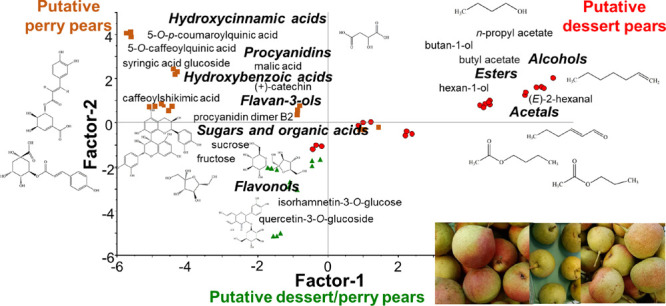

The phenolic profiles and other major metabolites in juices made from fruits of 17 cultivars and selections of European pears were investigated using UHPLC-DAD-ESI-QTOF-MS and GC-FID, respectively. A total of 39 phenolic compounds were detected, including hydroxybenzoic acids, hydroxycinnamic acids, flavan-3-ols, procyanidins, flavonols, and arbutin. Among these compounds, 5-*O*-caffeoylquinic acid was the most predominant, accounting for 14–39% of total quantified phenolic contents (TPA) determined in this study. The variations were mainly cultivar dependent. The genetic background effect on the chemical compositions is complex, and breeding selections from the same parental cultivars varied dramatically in chemical compositions. Putative perry pears contained more 4-*O*-caffeoylquinic acid, 5-*O*-caffeoylquinic acid, caffeoyl *N*-trytophan, caffeoylshikimic acid, coumaroylquinic acid isomer, syringic acid hexoside, procyanidin dimer B2, (+)-catechin, and malic acid, whereas putative dessert pears had higher esters, alcohols, and aldehydes. The results will be helpful in providing industry with phytochemical compositional information, assisting pear selections in commercial utilization.

## 1 Introduction

Pear (*Pyrus spp*.) fruit is the fifth most widely cultivated fruit in the world. The annual production of pears is approximately 23.1 million tons globally in 2020, of which 2.8 million tons was produced in Europe and mainly consisted of European (Occidental) pear (*P. communis* L.).^1^ Contrary to the crispy Asian pears (e.g., *P. pyrifolia* Nakai, *P. ussuriensis* Maxim, and related hybrids), the European pears typically have a soft and smooth flesh texture.^2^ Unavoidably, a large amount of pear fruits are wasted annually, as they do not reach the fresh markets due to the low fruit quality or logistical issues. Approximately 45% of the global fruit and vegetable production is lost yearly.^1^ The losses of pear fruits may be ascribed to the high temporal and local variation at the farm (5–25%) and storage (8–29%) levels in the fruit supply chain.^3^ The juiced fraction, e.g., fruits with external defects, low internal quality, or a wrong maturation time for a target market, still contains high nutritional value with notable amounts of sugars, minerals, amino acids, and phenolic compounds.^4^ They also have higher levels of dietary fibers but lower calorie contents than some of the most common fruits and vegetables, as previously reported.^5,6^ Thus, from the points of view of commerce and sustainability, it is essential to transfer the wasted fruits into value-added products, such as fresh juices, canned jellies, canned jams, alcoholic beverages, and dry fruits.^7−9^

The Nordic countries produce 7 million kg of pear in total, less than 1% of the European pear production.^1^ For example, in Finland, the long history of local and home garden pear cultivation has not survived to modern retail supply chains. The old cultivars have a short storage and shelf life or low fruit quality. Unfortunately, only a few of the European commercial cultivars can be grown in the climatically favorable South-Western Finland.^10^ Variable weather conditions increase the risk of yield and quality losses despite the warming climate.^11^ Adapted cultivars that are bred for hardiness and for multiple fruit uses are a sustainable option for fruit production in Northern Europe because they ensure more stable income for growers. Characterization of the fruits of prospective new cultivars for alternative or main use in beverage production (pear juice or perry) is therefore important for reducing fruit loss and waste.

Fruit quality can be described as the combination of organoleptic and nutritional aspects tightly correlated with the bioactive compounds as well as shelf life, fruit size, and juiciness, which are highly dependent on the cultivar. New pear cultivars can be bred by crossing two cultivars and selecting new cultivar candidates from their offspring. However, hidden genetic variation in the parent cultivars may result in unexpected variation of fruit quality traits in the breeding progenies. In general, dessert pears are popular due to their pleasant taste, high nutritional properties, and good storability, whereas perry pears are smaller, bitter, and more astringent with high concentrations of polyphenols.^6,12^ For example, “Fausset”, “De Cloche”, and “Plant de Blanc” are used as perry pears, whereas “Conference” and “Williams” are widely used as dessert pears in European countries.^6^ Phenolic compounds were reported to influence the sensory properties of fruits positively or negatively, especially the color, flavor, and astringency.^13^ In pear juices, the predominant polyphenolic constituents are mainly hydroxycinnamic acids, including caffeic acid, *p*-coumaric acid, ferulic acid, and their derivatives.^6,14^ Among these compounds, chlorogenic acid has been reported to be the dominant phenolic acid in the pear juices made from five Australian-grown pear varieties.^9^ A number of flavan-3-ols, procyanidins, and flavonol glycosides were also found in pear fruits, as well as simple phenolics, such as arbutin.^15^ In addition, the sugar/acid ratio varied among pear cultivars and significantly affected the sour and sweet taste of fruits. No significant differences in total quantified sugar contents were found between apples and pears, whereas the total quantified organic acid contents of pears were significantly lower than those of apples.^16^ Pear has been reported to contain the highest amount of sorbitol among certain fruit juices (apple, pear, peach, grape, sweet cherry, strawberry, and blueberry).^16^ Aroma compounds also played an important role in affecting overall flavor of pear fruits, which determined the consumer perception and acceptability of the final pear products. Esters and alcohols were detected as the main aroma compounds as reported in European (Occidental) pears and Asiatic (Oriental) pears, such as “*Niitaka*” (*P. pyrifolia)* and Korla pear (*P. bretschneideri* Rehd.).^17−19^

The main aim of the present study was to characterize the chemical profiles of pear juices made from fruits of breeding selections and test cultivars and to investigate their potential for juice and perry uses. Two commercial dessert pear cultivars (“Conference” and “Clara Frijs”), with pleasant flavor, juiciness, and aroma, were included as external standard cultivars. Moreover, “Conference” is the most commonly grown cultivar and one of the most important produced fruits in European countries.^20^ In the current work, qualitative and quantitative analyses of pear phenolic compounds, including phenolic acids, flavan-3-ols, procyanidins, flavonols, and arbutin (hydroquinone), were conducted with ultrahigh-performance liquid chromatography equipped with a diode array and an electrospray quadrupole/time-of-flight tandem mass spectrometer (UHPLC-DAD-ESI-QTOF) and UHPLC-DAD. To the best of our knowledge, studies on the variability of phenolic composition among pear juices produced from fruits of different cultivars are scarce in the current literature. This is also the first report to characterize the range of variation in the phenolic profiles and composition among breeding selections from controlled crosses between dessert cultivars. Moreover, the profiles of sugars, organic acids, and main volatiles related to the overall quality of pear juices were also characterized by using a gas chromatograph equipped with a flame ionization detector (GC-FID). Multivariate models, including principal component analysis (PCA) and partial least squares-discriminant analysis (PLS-DA), were also applied to study the relationships between the key chemical variables and the samples and/or sample grouping developed by breeders. The genetic background effect has been also considered in the study, making this study a significant starting point for future investigations on the heritability and genetic determinants of fruit biochemical composition and its variation available in European pear. The findings of this study can help breeders in the more targeted application of biochemical analyses in selection of new cultivars and meeting the breeding targets of fruit quality suitable for multiple or additional use, such as juice and perry making. In addition, the current study provides the fruit industry with important compositional information on phytochemicals, assisting in the selection of pear cultivars for commercial utilization.

## 2 Materials and Methods

### 2.1 Chemicals

LC and LC–MS grade chemicals were purchased from VWR International Oy (Espoo, Finland). Ethanol (≥99.7%) was purchased from Altia Oyj (Helsinki, Finland). The standards of ethyl acetate, acetaldehyde, butan-1-ol, and acetic acid were obtained from Sigma-Aldrich (St. Louis, MO, United States). The standards of myo-inositol, xylose, fructose, glucose, sorbitol, sucrose, tartaric acid, malic acid, succinic acid, citric acid, quinic acid, and ascorbic acid were purchased from Extrasynthese (Genay, France). (−)-Epicatechin, (+)-catechin, arbutin, 3-*O*-caffeoylquinic acid, 5-*O*-caffeoylquinic acid, *p*-coumaric acid, gallic acid-4-*O*-glucoside, and caffeic acid were provided by Sigma-Aldrich Co. (St. Louis, MO, United States). Quercetin-3-*O*-glucose, kaempferol-3-*O*-glucose, and procyanidin B2 were obtained from Extrasynthese (Genay, France).

### 2.2 Plant Materials and Sample Preparation

Seventeen samples of pear fruits were included in the study, including fruits of two commercial cultivars (“Conference” and “Clara Frijs”), two test cultivars (“Stolishnaia” and “Krupnoplodnaja Susova”), and 13 unreleased breeding selections from the pear breeding program of Natural Resources Institute Finland (Luke) (Table 1). The breeding selections were selected from the progenies of six controlled crosses between cultivars of European pear (*Pyrus communisL.*) (Table 1), and they represent the variation in fruit quality available in the breeding germplasm. The test cultivars that are not in commercial fruit production in Finland, “Stolishnaia” (Sto) and “Krupnoplodnaja Susova” (Kru), have been developed by Moscow Timiryazey Agricultural Academy (Russia) and are being observed for climatic adaptation and suitability for the juice market. Fruits of the breeding selections and the two test cultivars were produced in the experimental orchard of Luke in Piikkiö, Kaarina, Finland (60°39′N, 22°55′E; 18 m asl), in 2019. The fruits were collected at harvest maturity, as determined by flesh firmness, seed color, taste, and abscission. The trees were 8–10 years old and were grown in a trellis support system and grafted onto *P. communis* seedling rootstock (two test cultivars) or supported by their own roots (13 breeding selections). The trees were drip irrigated and trained in a modified central leader with a tree spacing of 3.5 × 1 m. The ground at the tree rows was covered with woven polypropylene groundcover (MyPex), and the inter-row spaces were covered by grass. Two chemical control sprays against scab (caused by *Venturia* spp.) were applied during the season. Fertilization was distributed on the ground surface or added via drip irrigation, and the amount and product selection were calculated based on nutrient requirements as supported by the soil test. Additionally, pear fruits of the cultivar “Conference” (Con) produced in the Netherlands and “Clara Frijs” (Cla) produced elsewhere in southern Finland were purchased from the local supermarket (Table 1).

**Table 1:** Description of the Pear Cultivars and Pear Selections in the Study and Their Parent Cultivars

sample code	cultivar or breeding	name of cultivar or cross^a^	harvest date	fruit description of overall sensory impression^b^	tentative use by breeders^c^
Py1	selection	Pepi × Lück	2019/8/30	sweet, astringent, mild aroma, and spicy	C
Py2	selection	Pepi × Lück	2019/8/30	mild sweetness and mild aroma	D
Py3	selection	Pepi × Lück	2019/8/30	acidic, mild astringent and bitter	C
Py4	selection	Pepi × Lück	2019/9/12	sweet and aromatic	D
Py5	selection	Pepi × Lück	2019/9/4	acidic, astringent, and aromatic	C
Py6	selection	Alna × Lück	2019/8/29	sweet, acidic, aromatic, juicy, and mild astringent	D/C
Py7	selection	Alna × Lück	2019/8/29	sweet, aromatic, juicy, and mild acidity; and astringent in peel	D/C
Py8	selection	Pepi × Pakurlan Päärynä	2019/9/12	sweet, highly aromatic, and mild astringent	D/C
Py9	selection	Karmla × Pakurlan Päärynä	2019/9/12	sweet, acidic, and highly aromatic	D
Py10	selection	Karmla × Pakurlan Päärynä	2019/8/30	sweet, astringent, bitter, and spicy	C
Py11	selection	Rumnaja Kedrina × Pakurlan Päärynä	2019/8/30	mild sweetness, mild astringent, and mild aroma	D/C
Py12	selection	Rumnaja Kedrina × Pakurlan Päärynä	2019/8/30	astringent and mild bitterness	C
Py13	selection	Lukna × Pakurlan Päärynä	2019/8/29	sweet, aromatic, and mild astringent	D
Sto	test cultivar	Stolishnaja/Stolichnaya	2019/9/10	acidic, astringent, and juicy	C
Kru	test cultivar	Krupnoplodnaja Susova	2019/9/19	juicy	D
Con	cultivar	Conference			
Cla	cultivar	Clara Frijs			

a The cross is expressed by “maternal cultivar × pollen cultivar”: the parental cultivars “Pepi”, “Lück”, “Pakurlan Päärynä”, and “Rumnaja Kedrina” are originated from Estonia, German, Finland, and Russia, respectively, whereas “Alna”, “Karmla”, and “Lukna” are originated from Latvia. Two test cultivars “Stolishnaja /Stolichnaya” and “Krupnoplodnaja Susova” are originated from Russia. The commercial dessert pear (used as references) cultivar “Conference” is of British origin and “Clara Frijs” of Danish origin, were purchased from the local supermarket.

b Fruit description of the obtained pear selections, and descriptors of the test pear cultivars were determined by an in-house panel of the pear breeding program.

c The tentative uses of breeding selections (from “Py1” to “py13”) and test cultivars (“Sto” and “Kru”) were divided into dessert pears (D) and juice/perry pears (C); the two classifications were determined by an in-house panel of pear breeding program and based on the sweetness and astringency of fruits at their eating ripeness. In addition, commercial cultivars (“Con” and “Cla”) were divided into dessert pears (D) as they are sold as commercial pears in the supermarket.

The fruit samples were stored in a fruit storage chamber (+1 – +3 °C, RH > 90%, ventilation) and assessed for fruit quality and maturity. At the eating ripeness of each sample, fruits for juicing were carefully selected for fruit size, maturity, and absence of external or internal damage. The fruits were washed, sliced into small pieces, and then crushed into juices with a juice presser. For each cultivar, the juices were pressed in triplicate and stored at −20 °C immediately until further chemical analyses.

### 2.3 Analysis of Phenolic Compounds

Extraction of phenolic compounds was carried out according to the method reported by a previous study with slight modifications.^21^ Briefly, phenolics were extracted from pear juices (25 mL) with 20 mL of ethyl acetate, assisted by sonication (20 min) and centrifugation (4500 × *g*, 15 min) for four times. All the supernatants were combined and evaporated at 35 °C until completely dry. The residue was dissolved in 1.5 mL of methanol and filtered through a 0.2 μm PTFE filter before injection.

Liquid chromatography separation was performed according to a published method with slight modifications.^22^ The identification was performed via a UHPLC-DAD-ESI-QTOF system (Bruker Daltonik GmbH, Germany) consisting of a Bruker ultrahigh-performance liquid chromatograph (UHPLC) in combination with an Elute diode-array detector (DAD), an electrospray ion (ESI) source, and an Ultra-High Resolution Impact II quadrupole/time-of-flight (Q-TOF) tandem mass spectrometer. The column used was an Aeris peptide XB-C18 column (150 × 4.60 mm, 3.6 μm) from Phenomenex (Torrance, USA). Mobile phase A consisted of 0.1% formic acid in water, and mobile phase B consisted of 0.1% formic acid in acetonitrile. The gradient used was as follows: 2–4% B, 0–5 min; 4–7% B, 5–10 min; 7–8% B, 10–15 min; 8–10% B, 15–20 min; 10–18%, 20–30 min; 18–20% B, 20–35 min; 20–25% B, 35–40 min; 25–35% B, 40–45 min; 35–40% B, 45–46 min; 40–70% B, 46–49 min; 70–2% B, 49–51 min; 2% B, 51–53 min, sequentially. The flow rate was 1.0 mL/min at 25 °C. After splitting, 0.4 mL/min of LC eluent was directly flown into the MS system. The mass spectrometer was operated under both negative and positive ion modes with the following source settings: end plate offset, 500 V; nebulizer gas pressure, 2.5 bar; drying gas flow, 11 L/min; drying gas temperature, 280 °C; capillary voltage, 4.5 kV (positive mode) and 3.5 kV (negative mode); quadrupole ion energy, 5.0 eV. The mass was scanned across the range of *m*/*z* 20–2000, and the range of collision energy was set as 5.0–12.5 eV. A sodium formate solution (10 mM) was continually introduced to the system as internal calibration for high-accuracy mass calibration. The MS data were collected and analyzed by Compass Data analysis software 4.4 (Bruker Daltonik GmbH, Germany).

Quantification of phenolic compounds with authentic standards was performed in a Shimadzu UHPLC-DAD system (Shimadzu Corp., Kyoto, Japan) using 5-*O*-caffeoylquinic acid, (+)-catechin, procyanidin B2, and quercetin-3-*O*-glucoside. Flavan-3-ols and hydroxybenzoic acids were recorded at 280 nm; hydroxycinnamic acids were recorded at 320 nm; and flavonols were recorded at 360 nm. The quantification was calculated using external standards. (+)-Catechin and procyanidin B2 were used to quantify monomeric flavan-3-ols and procyanidins, respectively. 5-*O*-Caffeoylquinic acid and gallic-4-*O*-gllucoside were used to quantify hydroxycinnamic acids and hydroxybenzoic acids, respectively, and quercetin-3-*O*-glucoside was used to quantify flavonols. Moreover, arbutin was also employed as an external standard to quantify the concentration of arbutin. The list of external standard curves is shown in Table S1.

### 2.4 Measurements of pH Values, Total Soluble Solids (°Brix), and Color Parameters

The pH value of the obtained juices was monitored by a pH meter (Weilheim, Germany). Total soluble solids (°Brix) were measured by a portable °Brix meter (Atago Co. Ltd., Tokyo, Japan). An Evolution UV–visible 300 spectrophotometer (Thermo Fisher Scientific, USA) was used for color measurements. Samples were analyzed in a 1 cm path length quartz cell at absorbances of 420, 520, and 620 nm. The color intensity was calculated as the sum of three absorbances (420, 520, and 620 nm), and the tonality (hue) was determined by calculating the ratio between the absorbance of 420 and 520 nm.^23^ The juice yields were calculated by dividing the volume of juice collected (mL) by the mass of pear fruit sample (kg). All the samples were centrifuged at 3000 × *g* for 10 min to remove precipitates before chemical analysis.

### 2.5 Measurements of Sugars and Organic Acids

Individual sugars and organic acids were analyzed as trimethylsilyl (TMS) derivatives by the GC-FID method based on our previous method with slight modifications.^24^ A gas chromatograph instrument together with a flame ionization detector (GC-FID, Shimadzu, Japan, model GC-2010plus) was used in the study. An SPB-1 column (30 m × 0.25 mm i.d., 0.25 μm) was used as the column. Myo-inositol and tartaric acid were used as internal standards of sugars and organic acids, respectively. The studied compounds were identified by comparing the retention times with those of the authentic standards. The total quantified contents of sugars and organic acids were expressed as the sum concentration of quantified individual sugar and organic acid compounds. The total sweetness index (TSI) was calculated by multiplying the average amounts of each sugar (sucrose, glucose, and fructose) and their relative sweetness respect to sucrose based on the following equations:
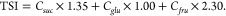
where *C_suc_*, *C_glu_*, and *C_fru_* are the average amounts of sucrose, glucose, and fructose, respectively. The contribution of each carbohydrate was calculated according to the assumption that the sweetness of fructose and sucrose are 2.30 and 1.35 times sweeter than glucose.^25^

### 2.6 Analysis of Major Volatile Compounds

The GC-FID method of volatile compound analysis was optimized based on our previous study.^22^ The same GC-FID system was used for the analysis of major volatiles, and an HP-INNOWax column (30 m × 0.25 mm, i.d., 0.25 μm) column was used in the study. The temperature program started from 40 °C and was held for 8 min; then, the temperature was increased to 240 °C (10 °C/min) and then held at 240 °C for 2 min. The injector temperature was at 220 °C, and the samples were injected automatically (1 μL) with a split ratio of 1:25. The detector temperature was set at 280 °C. The carrier gas was helium with a flow rate of 1 mL/min. All the samples were filtered through 0.2 μm PTFE filters before injection. The contents of major volatile compounds were determined with standard curves of ethyl acetate, butan-1-ol, acetaldehyde, and acetic acid. The external standard curves are shown in Table S1.

### 2.7 Statistical Analysis

The results are presented as the mean values ± standard deviation of triplicate observations. Statistical analysis was performed via SPSS 27 (IBM SPSS Statistics, Inc., Chicago, IL, United States). Significant differences among samples were determined using one-way ANOVA and Tukey’s test. Principal component analysis models of full-cross validation (including PCA and PLS-DA) were used to investigate the correlations between chemical compositions and different pear cultivars. To visualize the bivariate correlations between the selected chemical compounds (selected based on PCA analysis), a supervised hierarchical clustering analysis based on Pearson’s correlation coefficient was used via online software MetaboAnalyst 5.0 (McGill University, Canada).

## 3 Results and Discussion

### 3.1 Physicochemical Characteristics of Pear Cultivars

The juice yield, pH values, total soluble solids (°Brix), color tonality, and color intensity were analyzed from the processed juices obtained from 17 different pear cultivars and breeding selections (Table S2). The highest juice yields were found in the two test cultivars “Sto” (84%) and “Kru” (81%), which might be preferred by the juice-pressing industry based on economic and practical concerns. In addition, selection “Py7” from the breeding program also contained a high juice yield of 75%. The highest values were found in the cultivar “Con” (pH 4.6) and the cultivar “Cla” (pH 4.5), whereas the lowest values were found in “Sto” (pH 3.3) and “Kru” (pH 3.7). According to previous reports,^21^ pH was used to indicate the sourness when evaluating different fruit juices and fruit wines. The juice made from the pear selections derived from the breeding program had lower pH in general, indicating that these developmental pear cultivars were sourer than the studied dessert cultivars. Apart from the pH values, the °Brix value has been reported to determine the internal quality attributes and is an important indicator of soluble single sugars or organic acids. In general, fruit juices with higher °Brix values are perceived sweeter and consequently appreciated by consumers. The pear cultivar “Py8” showed the highest °Brix value (16.7) whereas the cultivar “Sto” showed the lowest °Brix value (8.5). Interestingly, the pear selections of the breeding program sharing same parental cultivars (Table S2) did not share similar physiological characteristics, as expected by the complex inheritance and low or moderate heritability of these traits in pear.^26−28^

### 3.2 Identification of Chemical Compounds in Pear Juices

In the present work, the identification analysis of phenolic compounds in 17 pear cultivars was conducted by UHPLC-DAD-ESI-QTOF in both positive and negative ionization modes. Altogether, 39 phenolic compounds were identified via the comparison of the retention times, UV–vis spectra, and mass spectra with those of the reference compounds and the results reported in the literature,^9,15,29−35^ primarily as hydroxybenzoic acids (3 compounds), hydroxycinnamic acids (18 compounds), monomeric flavan-3-ols (2 compounds), procyanidins (5 compounds), flavonols (10 compounds), and arbutin. The qualitative results and LC chromatograms are shown in Table 2 and Figure S1, respectively. As shown in Table 2, different hydroxycinnamic acids were detected in the studied pear juices, including derivatives of caffeic, coumaric, ferulic, and sinapic acids as well as free caffeic and *p*-coumaric acid. For the hydroxybenzoic acids, only syringic acid and its glycosylated derivatives were detected. In the flavan-3-ol group, (+)-catechin and (−)-epicatechin were detected by comparing their retention times, UV–vis, and MS spectra with those of reference standards. The procyanidins in the studied pear juices were identified mostly as B-type procyanidins. A dimer of A-type procyanidin was also detected in the study. Flavonols were identified as glycosides of quercetin, kaempferol, and isorhamnetin.

**Table 2:** Identification of Phenolic Acids, Flavan-3-Ols, Procyanidins, Flavonols, and Other Phenolic Compounds in the Studied Pear Juices^a^

			measured mass (*m*/*z*)	theoretical mass (*m*/*z*)	mass error (ppm)				
peak	tentative identification	λ_max_ (nm)	[M – H]^−^/[M + H]^+^	[M – H]^−^/[M + H]^+^	[M – H]^−^/[M + H]^+^	negative ions in MS^2^ (*m*/*z*)	positive ions in MS^2^ (*m*/*z*)	molecular formula	identification method
Hydroxybenzoic Acids									
3	syringic acid hexoside I	275	359.0954/–	359.0978/–	7.18/–	197.0476		C_15_H_20_O_10_	MS and literature^15^
8	syringic acid	277	197.0444/199.0598	197.0450/199.0606	3.04/4.01			C_9_H_10_O_5_	MS and literature^31^
9	syringic acid hexoside II	274	359.0963/–	359.0978/–	4.18/–	197.0472		C_15_H_20_O_10_	MS and literature^15^
Hydroxycinnamic Acids									
13	caffeoyl *N*-tryptophan	326	365.1332/–			229.4809			MS and literature^29,36^
14	3-*O*-caffeoylquinic acid	327	353.1003/355.0992	353.0872/355.1028	–4.29/9.92	191.0565		C_16_H_18_O_9_	MS, standard, and literature^29,30,33^
15	5-*O*-caffeoylquinic acid	328	353.0875/355.0993	353.0872/355.1028	–0.85/9.86	191.0563	163.0353	C_16_H_18_O_9_	MS, standard, and literature^9,29,30,33^
16	caffeic acid	319	179.0344/181.0495	179.0345/181.0501	0.56/3.31			C_9_H_8_O_4_	MS, standard, and literature^29,33^
17	4-*O*-caffeoylquinic acid	327	353.0869/355.0994	353.0872/355.1028	0.85/9.57	173.0446	163.0362	C_16_H_18_O_9_	MS and literature^29,30,33^
18	*p*-coumaric acid	301	163.0407/165.0546	163.0395/165.0546	–4.29/0.00			C_9_H_8_O_3_	MS, standard, and literature^29^
19	coumaroylquinic acid isomer I	320	337.0915/339.1047	337.0923/339.1079	2.37/9.43	173.0445	147.0414	C_16_H_18_O_8_	MS and literature^9,29,30^
20	sinapic acid hexoside I	320	385.1121/387.1319	385.1135/387.1291	3.64/–7.23	223.0577	235.0218	C_17_H_22_O_10_	MS and literature^29^
21	caffeoylshikimic acid	326	335.0751/337.0896	335.0767/337.0923	4.78/8.01	179.0343	163.0356	C_16_H_16_O_8_	MS and literature^32^
22	coumaroylquinic acid isomer II	318	337.0907/339.1059	337.0923/339.1079	4.75/5.90	191.0553	147.0408	C_16_H_18_O_8_	MS and literature^29,30^
23	di-*O*-caffeoylquinic acid I	327	515.1172/517.1323	515.1190/517.1346	3.49/4.02	353.0879/191.0553		C_25_H_24_O_12_	MS and literature^33,36^
24	di-*O*-caffeoylquinic acid II	327	515.1173/517.1325	515.1190/517.1346	3.30/4.06	353.0881/191.0552		C_25_H_24_O_12_	MS and literature^33,36^
25	feruloylquinic acid isomer I	327	367.1012/369.1169	367.1029/369.1185	4.63/4.33	193.0513		C_17_H_20_O_9_	MS and literature^30^
26	feruloylquinic acid isomer II	327	367.1013/369.1174	367.1029/369.1185	4.60/2.98	193.0514, 173.0341	163.0352	C_17_H_20_O_9_	MS and literature^30^
27	coumaric acid derivative	313	581.0865/583.0992			279.0504, 163.0397	303.0432, 147.0398		MS and literature^34^
28	ferulic acid derivative	324	389.1451/391.1620	389.1448/391.1604	0.77/4.09	193.0496			MS
29	sinapic acid hexoside II	320	385.1141/387.1259	385.1135/387.1291	1.56/–8.26	223.0571	235.0223	C_17_H_22_O_10_	MS and literature^29^
30	caffeoylhexose	308	341.0852/343.1032	341.0873/343.1029	–6.16/0.87	191.0338, 179.0349	265.0819, 307.0921	C_15_H_18_O_9_	MS and literature^36^
Flavan-3-ols									
5	(+)-catechin	283	289.0760/291.0832	289.0712/291.0868	–7.26/10.06	245.0800, 203.0700	207.0625, 139.0370	C_15_H_14_O_6_	MS, standard, and literature^36^
7	(−)-epicatechin	278	289.0705/291.0852	289.0712/291.0868	2.42/5.49			C_15_H_14_O_6_	MS, standard, and literature^36^
Procyanidins									
2	A type procyanidin dimer	279	575.1179/–	575.1192/–	–2.26/–	449.0859, 289.0709		C_30_H_24_O_12_	MS and literature^9,36^
4	B type procyanidin dimer I	279	577.1302/579.1463	577.1346/579.1502	7.15/6.52	407.0628, 289.0696, 161.0266	427.0994, 409.0923, 291.0682, 247.0692, 163.0417	C_30_H_26_O_12_	MS and literature^9,36^
6	procyanidin dimer B2	280	577.1305/579.1465	577.1346/579.1502	7.10/6.39	407.0633, 289.0690, 161.0287	427.0991, 409.0915, 291.0680, 247.0688, 139.0360	C_30_H_26_O_12_	MS, standard, and literature^36^
10	B type procyanidin trimer	280	865.1917/867.2171	865.1980/867.2136	7.28/–4.04		579.1326, 409.0872, 291.0875,163.0349	C_45_H_38_O_18_	MS and literature^9,29^
11	B type procyanidin dimer II	280	577.1304/579.1465	577.1346/579.1502	7.13/6.39	407.0711, 289.0691, 161.0235	427.0982, 409.0913, 291.0789, 271.0639, 247.0698, 163.0411	C_30_H_26_O_12_	MS and literature^29^
Flavonols									
31	quercetin hexoside deoxyhexoside I	353	609.1423/611.1561	609.1456/611.1612	5.42/8.34	301.0256	303.0471, 465.0986	C_27_H_30_O_16_	MS and literature^29,36^
32	quercetin hexoside deoxyhexoside II	353	609.1420/611.1565	609.1456/611.1612	5.90/7.69	301.0260	465.0986	C_27_H_30_O_16_	MS and literature^29,36^
33	quercetin hexoside	348	463.0849/465.0985	463.0877/465.1033	6.05/10.32	301.0258	303.0467	C_21_H_20_O_12_	MS and literature^29^
34	quercetin-3-*O*-glucoside	352	463.0857/465.0982	463.0877/465.1033	4.32/10.63	301.0258	303.0473	C_21_H_20_O_12_	MS, standard, and literature^29^
35	isohamnetin hexoside deoxyhexoside	352	623.1572/625.1722	623.1612/625.1768	6.42/7.36	315.0492	317.0663	C_28_H_32_O_16_	MS and literature^29^
36	kaempferol-3-*O*-glucoside	352	447.0907/449.1047	447.0928/449.1084	4.70/8.24	285.0313	287.0523	C_21_H_20_O_11_	MS, standard, and literature^29^
37	isorhamnetin hexoside I	352	477.1008/479.1147	477.1033/479.1189	5.24/8.77	315.0417	317.0634	C_22_H_22_O_12_	MS and literature^29,36^
38	isorhamnetin hexoside II	352	477.1021/479.1145	477.1033/479.1189	2.52/9.18	315.0428	317.0632	C_22_H_22_O_12_	MS and literature^29,36^
39	isorhamnetin-acylated-hexoside I	352	519.1101/521.1255	519.1139/521.1295	7.32/7.67	315.0495		C_24_H_24_O_13_	MS and literature^29,36^
40	isorhamnetin-acylated-hexoside II	354	519.1111/521.1255	519.1139/521.1295	5.39/7.67	315.0493		C_24_H_24_O_13_	MS and literature^29,36^
Other Phenolics									
1	arbutin	282	271.0812/273.0958	271.0818/273.0974	2.21/5.86	109.0964		C_12_H_16_O_7_	MS and literature^9^
12	unknown	275	265.1475/–						MS

a The peak numbers in the table refer to those in Figure S1.

### 3.3 Content of Phenolic Compounds in Pear Juices

The phenolic profiles of pear juices made from 17 cultivars are summarized in Table 3. Phenolic compounds have been reported to be responsible for the astringency and provide proper taste in fruit beverages with a concentration range of 300–800 mg/L.^37^ The total quantified phenolic contents (TPA, calculated as the sum of individual phenolic compounds) of the studied pear juices ranged from 172.9 mg/L in cultivar “Con” to 714.6 mg/L in cultivar “Py10”. The variation in phenolic profiles might be explained by the different genetic backgrounds and maturity levels of fruit cultivars.^38^ The test cultivar “Sto” (tentatively used as juice/perry pears) contained a high TPA of 654.3 mg/L, whereas lower TPA were found in the dessert pear groups, such as the test cultivar “Kru” (177.5 mg/L) as well as the two commercial dessert cultivars “Con” (172.9 mg/L) and “Cla” (209.7 mg/L). Pear breeding selections sharing the same parental cultivars did not always have similar total quantified phenolic contents. For example, the TPA of the pear selections derived from the cross “Pepi × Lück” ranged from 335.4 (“Py4”) to 688.9 mg/L (“Py2”), whereas two pear selections (“Py6” and “Py7”) derived from the cross “Alna × Lück” shared similar TPA. Thus, the genetic effect on the phenolic profiles of pear is complex, as already indicated by a moderate heritability of skin bitterness.^25^

**Table 3:** Concentrations of Individual Phenolic Compounds and Major Phenolic Groups in Pear Juices (mg/L)^a^

pears	3	8	9	TBA	14	13	15	16	17	18	19	20	21	22	23	24	25
Py1	30.85	0.97	14.49	48.68	ND	6.32	195.41	3.64	26.25	4.17	7.67	ND	12.37	14.84	0.99	1.13	14.51
Py2	28.82	ND	12.16	79.38	2.96	8.01	184.31	ND	22.95	ND	9.18	11.39	8.81	10.25	0.93	0.39	27.67
Py3	11.76	ND	55.90	75.53	3.20	2.85	201.44	6.18	36.54	1.03	1.03	2.57	3.06	3.03	ND	ND	ND
Py4	19.98	ND	11.36	31.34	4.06	3.21	79.76	1.00	24.36	2.70	4.03	2.81	ND	3.13	0.82	ND	8.51
Py5	17.12	ND	30.42	49.30	3.96	0.21	155.63	2.74	19.09	1.27	20.03	3.92	2.23	3.38	1.36	ND	18.34
Py6	13.53	1.94	4.70	20.17	ND	4.05	158.66	1.03	20.42	0.88	4.30	5.33	2.09	6.25	ND	ND	ND
Py7	6.14	ND	17.88	29.49	2.18	3.91	155.40	3.05	24.94	0.86	8.76	9.04	5.12	4.17	6.57	ND	18.87
Py8	35.50	ND	12.94	59.62	2.62	1.41	149.61	0.67	18.47	ND	12.49	2.89	7.18	6.98	ND	ND	11.06
Py9	8.76	ND	15.20	36.30	5.90	2.09	117.74	1.03	16.01	0.22	2.14	4.39	2.03	2.97	0.98	4.25	ND
Py10	27.37	5.02	16.81	99.12	5.47	2.09	200.68	3.04	37.61	1.00	25.93	3.14	9.05	10.22	0.46	4.11	21.46
Py11	23.58	ND	16.96	40.54	8.48	0.53	161.13	ND	17.46	1.00	8.75	1.05	ND	ND	ND	1.48	8.80
Py12	23.80	ND	11.30	35.10	4.52	0.27	51.05	0.98	7.29	0.74	ND	ND	ND	ND	ND	ND	ND
Py13	16.92	ND	6.10	23.02	ND	0.12	37.05	ND	3.24	2.10	ND	ND	ND	ND	ND	ND	ND
Sto	17.22	5.76	91.41	127.22	0.99	1.33	217.39	5.09	30.61	ND	41.36	7.56	ND	1.12	5.68	ND	31.65
Kru	7.99	ND	11.90	24.65	3.89	ND	26.52	ND	4.39	0.32	7.44	ND	ND	ND	ND	ND	7.61
Con	7.28	ND	11.60	18.88	2.25	ND	38.67	ND	2.90	0.93	3.10	ND	ND	ND	ND	ND	1.48
Cla	2.52	ND	28.37	30.90	3.19	ND	36.88	ND	1.97	1.02	3.07	ND	ND	ND	ND	ND	2.09

pears	26	27	28	29	30	TCA	5	7	TFA	2	4	6	10	11	TPY	31
Py1	3.16	1.33	0.92	17.14	0.88	304.41	20.75	11.61	32.36	2.38	7.61	15.79	12.49	10.60	46.48	ND
Py2	3.18	ND	2.44	ND	7.14	291.60	20.23	12.47	32.70	38.41	21.66	20.03	11.98	17.58	71.24	ND
Py3	2.56	0.45	ND	20.30	ND	281.38	12.49	75.12	87.61	7.87	9.00	27.25	2.01	23.78	62.04	0.62
Py4	ND	ND	ND	1.15	1.00	133.37	14.77	12.84	27.61	ND	2.91	17.93	10.51	28.40	59.75	ND
Py5	2.89	ND	ND	0.38	2.16	237.35	10.79	21.27	32.06	1.76	4.97	1.24	1.87	6.99	15.07	ND
Py6	5.36	6.12	ND	8.79	8.37	227.59	12.87	14.12	26.99	ND	3.67	25.47	10.94	22.83	62.91	0.38
Py7	5.40	5.45	ND	6.87	0.41	257.10	11.50	21.28	32.78	5.46	12.84	13.13	18.44	7.74	52.15	ND
Py8	0.65	ND	0.91	3.22	5.16	221.90	24.94	27.86	52.80	11.18	8.38	10.72	7.14	16.01	42.24	ND
Py9	4.98	0.76	ND	5.12	ND	168.51	9.72	12.66	22.38	12.34	6.75	15.75	11.77	13.75	48.03	0.77
Py10	5.54	2.57	ND	2.89	1.08	334.26	32.41	27.58	59.99	49.92	28.34	19.74	15.76	56.48	120.32	1.34
Py11	3.77	ND	ND	2.28	ND	214.21	16.43	44.16	60.59	ND	10.51	15.82	2.20	31.46	60.00	ND
Py12	4.15	2.94	ND	0.40	10.86	82.93	17.53	9.81	27.34	ND	15.04	12.29	11.95	18.26	57.53	ND
Py13	4.01	2.04	ND	0.34	1.11	49.90	10.66	10.27	20.94	ND	10.79	6.83	5.44	9.54	32.60	ND
Sto	2.53	2.66	2.26	6.97	ND	355.86	ND	40.46	40.46	12.84	11.77	ND	ND	23.90	35.68	3.87
Kru	1.58	2.09	ND	ND	0.97	54.81	7.20	4.65	11.85	4.76	5.53	2.51	3.14	11.27	22.45	0.65
Con	ND	ND	ND	ND	0.90	50.23	5.82	7.88	13.70	ND	8.68	ND	ND	29.21	37.89	ND
Cla	ND	ND	ND	0.32	ND	48.54	8.39	16.16	24.55	ND	7.05	7.72	ND	16.79	31.56	ND

pears	32	33	34	35	36	37	38	39	40	TFO	12	1	TPA
Py1	ND	3.08	4.66	4.46	1.11	0.85	2.72	1.70	5.30	23.87	24.59	10.68	552.38
Py2	ND	9.81	10.26	9.00	4.14	3.20	7.16	4.28	17.08	64.93	59.60	29.50	688.88
Py3	2.17	17.35	7.79	15.31	4.75	1.05	3.75	2.48	7.12	62.38	30.56	19.78	658.32
Py4	ND	5.97	7.34	6.51	1.21	1.01	3.14	0.99	5.80	31.96	26.09	9.97	335.38
Py5	1.01	8.22	7.81	13.42	6.96	2.03	4.77	2.26	8.45	54.94	36.73	16.30	443.81
Py6	ND	3.50	5.90	8.03	0.31	0.72	4.16	3.16	8.84	35.00	70.93	6.90	486.56
Py7	ND	10.42	7.23	15.66	5.07	2.16	3.13	2.10	5.88	51.65	28.31	11.82	494.49
Py8	ND	7.38	6.50	11.61	2.19	1.93	4.14	3.46	4.90	42.12	60.28	25.36	496.12
Py9	1.57	28.28	34.93	43.77	0.51	ND	2.87	9.06	15.25	137.02	38.60	5.35	469.52
Py10	2.28	6.67	9.08	8.03	10.83	0.63	4.07	4.42	10.62	57.98	18.58	9.08	714.55
Py11	ND	4.58	6.01	2.17	1.04	ND	1.95	2.14	9.20	27.09	23.36	5.65	406.93
Py12	ND	1.48	5.44	1.34	ND	ND	1.00	ND	ND	9.26	14.58	4.38	234.76
Py13	ND	3.49	6.11	8.64	0.96	2.54	2.20	2.00	7.58	33.52	32.33	9.64	198.00
Sto	5.78	9.84	14.87	19.89	0.65	0.90	0.70	2.17	5.07	63.74	34.12	18.00	654.28
Kru	1.02	7.12	9.66	8.44	0.92	1.02	2.22	0.88	4.89	36.79	23.67	3.33	177.54
Con	ND	6.87	9.86	3.15	3.11	3.39	3.65	4.63	2.19	36.84	8.91	6.42	172.87
Cla	ND	6.87	10.98	2.86	3.24	3.30	5.74	5.14	13.09	51.22	17.34	5.57	209.68

a Results are presented as the average of triplicates. TBA: total quantified hydroxybenzoic acids, TCA: total quantified hydroxycinnamic acids, TFA: total quantified flavan-3-ols, TPY: total quantified procyanidins, TFO: total flavonols, TPA: total quantified phenolic compounds. Abbreviations of pear cultivars and phenolic compounds refer to Table 1 and Table 2, respectively. Complete information with standard deviation and significant differences are shown in Table S3.

The contents of individual phenolic compounds in different pear cultivars also differed significantly (*p* < 0.05) from each other (Table S3). In terms of hydroxybenzoic acids, the test perry pear “Sto” contained the highest contents of total quantified hydroxybenzoic acids (TBA) at 127.2 mg/L, and the lowest content was found in the commercial dessert pear cultivar “Con” (18.9 mg/L). The TBA ranged from 48.5 (“Cla”) to 355.9 mg/L (“Sto”) in the studied pear juices. The primary hydroxycinnamic acid in the studied pear juices, 5-*O*-caffeoylquinic acid (peak 15), accounted for 15–40% of TPA. Tanriöven and Ekşi also found that 5-*O*-caffeoylquinic acid represented the main phenolic compound in the pear juices made from seven different types of Turkish pear cultivars.^37^ The two commercial dessert pear cultivars (“Con” and “Cla”) as well as the test dessert pear cultivar “Kru” contained lower concentrations of 5-*O*-caffeoylquinic acid in the current study, whereas the highest content of 5-*O*-caffeoylquinic acid was found in the test perry pear cultivar “Sto” (217.4 mg/L) followed by selection “Py3” derived from “Pepi × Lück” (201.4 mg/L) and selection “Py10” derived from “Karmla × Pakurlan Päärynä” (200.7 mg/L).

The total quantified flavan-3-ol monomer contents (TFA) ranged from 11.9 mg/L (“Kru”) to 87.6 mg/L (“Py3”) in the studied pear fruits. The predominant flavan-3-ol monomer detected in the studied pear juices could be either (+)-catechin or (−)-epicatechin, which was mainly dependent on the pear cultivars. For example, the test cultivar “Sto” contained a high amount of (−)-epicatechin, whereas no (+)-catechin was found in this cultivar. “Py3”, “Py5”, “Py7”, “Py11”, and “Cla” were also dominated by (−)-epicatechin, whereas the other cultivars contained slightly higher (+)-catechin or similar concentrations of (+)-catechin and (−)-epicatechin. A previous study also found that (−)-epicatechin was the predominant flavan-3-ol monomer in most of the European and Tunisian pear fruits except for certain cultivars, such as “Abate” and “Comice”.^6^ Procyanidins were detected as the predominant phenolic group in pear fruit, and the concentration can be up to over 90% of TPA, as previously reported in the European and Tunisian pear cultivars.^6,39^ However, the concentration of procyanidins in pear juices was much lower than that in the pear fruits, which can be ascribed to the higher retention of procyanidins by cell wall materials and the lower water solubility of procyanidins compared with other phenolic compounds. In addition, the detected amounts of procyanidins were also affected by the extraction method used. The total quantified procyanidins (TPY) ranged from 15.1 (“Py5”) to 120.3 mg/L (“Py10”) in the studied pear cultivars (Table 3). Cultivar “Py10” (juice/perry pear) showed the highest concentration of TPY at 120.3 mg/L, with 104.6 mg/L of procyanidin dimers and 15.8 mg/L of procyanidin trimers. Generally speaking, perry pears contained higher contents of procyanidins with high degrees of polymerization.^6^ However, the pear cultivar “Py5”, which was grouped as juice/perry type by breeders, was found to have low TPY. The reason might be the strong binding of procyanidins to the cell walls in the cultivar “Py5”.

In terms of flavonols, “Py12” contained only 9.3 mg/mL of TFO, whereas extremely high amounts of flavonols were found in “Py9” (137.02 mg/L). The differences in TFO were highly dependent on the pear cultivars. Among the studied pears, quercetin derivatives (27–50%) and isorhamnetin derivatives (11–49%) were found in high concentrations, whereas kaempferol derivatives (0–18%) were only detected in trace amounts. Arbutin was reported as a characteristic phenolic present in pear juices.^40^ The highest amount of arbutin (29.5 mg/L) was found in “Py2”, and the lowest (3.3 mg/L) was found in cultivar “Kru”.

### 3.4 Sugar and Organic Acid Contents of Pear Juices

The main nutrients and taste components of fruit juices, sugars, and organic acids contribute to the main soluble contents and the sensory characteristics of pear juices, such as sweetness, sourness, and bitterness.^16^ As shown in Table 2, the total quantified sugar contents (sum of individual sugars) ranged from 67.4 (“Sto”) to 152.6 g/L (“Py8”). Compared with the two commercial pear cultivars, the breeding selections for potential dessert use contained higher concentrations of total quantified sugars as expected for “Py5” (88.5 g/L), “Py10” (99.3 g/L), and “Py13” (83.7 g/L). In general, the individual sugar and total quantified sugar contents should correlate well together with the sweetness characteristics of the fruit pulp. However, the pear selections “Py3” and “Py12” (described as acidic and astringent by breeders) were detected with high amounts of total quantified sugars in the obtained pear juices, which could be ascribed to maturation of the fruit pulp before juice processing or a small sample size evaluated by the breeder’s panel. The most abundant sugar in the studied pear juices was fructose, which is in agreement with previously published reports.^16,41^ Fructose contents varied from 42.1 to 84.4 g/L, being the highest in “Py3” and “Py8”. The glucose concentration was found to be higher in “Py1” (30.5 g/L) than in the other cultivars. The highest level of sucrose was found in “Py8” (32.4 g/L) followed by “Py4” (30.2 g/L), “Py12” (28.8 g/L), and “Py6” (28.2 g/L). “Py12” showed the highest sorbitol content of 24.5 g/L among the studied pear juices. Xylose was detected in all the studied pear juices at trace amounts, ranging from 0.3 to 1.9 g/L.

Succinic acid, malic acid, quinic acid, citric acid, and ascorbic acid were the main organic acids identified from the pear juice samples (Table 4). The total quantified organic acids (sum of individual organic acids) varied from 3.6 to 11.3 g/L in the studied pears. “Py3” was found to contain the highest amount of total quantified organic acids (11.3 g/L), followed by “Sto” and “Py1” at concentrations of total quantified organic acids of 10.4 and 10.3 g/L, respectively. Malic acid was the most abundant organic acid in pear juices, as previously reported.^42^ Similar results were also found in the current study; the malic acid concentrations in the studied pear juices ranged from 1.8 (“Kru”) to 8.6 g/L (“Py3”). The content levels of quinic acid and citric acid were mainly dependent on the pear cultivars. Quinic acid was the second most abundant organic acid in most of the studied pear juices, whereas citric acid was the second most abundant organic acid in certain pear cultivars, such as “Py11” and “Py12” derived from “Rumnaja Kedrina × Pakurlan Päärynä”. A previous study also demonstrated that citric acid was the second most abundant organic acid in “Dangshan” pear juices.^41^ For the minor organic acids, all the studied juices were quantified with similar contents of succinic acid (0.2–0.5 g/L) and ascorbic acid (0.2–0.7 g/L).

**Table 4:** Concentrations of Quantified Sugars and Organic Acids in the Studied Pear Juices (g/L)^a^

pear	glucose	sucrose	fructose	sorbitol	xylose	sum of quantified sugars	*TSI* index	succinic acid	malic acid	quinic acid	citric acid	ascorbic acid	sum of quantified organic acids
Py1	30.53	21.57	69.02	20.08	0.99	142.18	218.40	0.54	7.83	1.32	0.24	0.34	10.26
Py2	24.81	16.10	61.88	9.21	0.51	112.51	188.87	0.23	6.11	1.41	0.13	0.33	8.21
Py3	24.27	17.74	84.37	14.93	0.89	142.19	242.27	0.49	8.61	1.84	0.12	0.25	11.31
Py4	20.75	30.23	69.12	14.41	0.86	135.37	220.54	0.40	4.42	1.55	0.62	0.46	7.45
Py5	14.69	11.40	54.56	6.37	1.44	88.46	155.57	0.37	5.88	1.12	0.19	0.29	7.85
Py6	15.28	28.23	68.84	13.26	0.72	126.34	211.72	0.44	4.75	1.34	0.13	0.31	6.97
Py7	13.57	24.34	54.21	13.69	0.44	106.25	171.12	0.43	4.73	0.75	0.15	0.27	6.33
Py8	17.06	32.35	83.27	18.81	0.53	152.56	252.25	0.39	6.87	1.11	0.15	0.31	8.84
Py9	10.15	20.42	62.61	13.19	0.43	106.80	181.72	0.38	4.02	1.46	0.14	0.22	6.21
Py10	8.80	22.51	52.72	13.56	1.66	99.25	160.44	0.37	5.11	1.17	0.20	0.43	7.28
Py11	14.14	19.17	59.59	19.19	1.85	113.93	177.08	0.32	3.53	1.26	1.46	0.41	6.97
Py12	16.13	28.82	62.54	24.52	0.92	132.93	198.88	0.33	2.95	1.38	1.46	0.39	6.51
Py13	10.76	6.33	52.25	13.27	1.05	83.65	139.48	0.39	2.50	1.72	0.27	0.37	5.26
Sto	7.38	8.63	42.10	8.17	1.16	67.44	115.86	0.43	7.22	1.36	1.14	0.29	10.44
Kru	10.95	10.33	47.47	11.42	0.72	80.89	134.08	0.38	1.83	1.00	0.03	0.40	3.61
Con	15.42	11.14	52.73	17.93	1.29	98.50	151.74	0.27	2.73	1.16	0.08	0.49	4.73
Cla	18.01	13.72	47.63	23.09	0.26	102.71	146.08	0.33	3.16	1.13	0.20	0.70	5.51

a Results are presented as the average of triplicates. Abbreviations of pear cultivars refer to Table 1. The compound codes relate to the sugars and organic acids were followed as: glucose (41), sucrose (42), fructose (43), sorbitol (44), xylose (45), succinic acid (46), malic acid (47), quinic acid (48), citric acid (49), and ascorbic acid (50). Complete information with standard deviation and significant differences are shown in Table S4.

### 3.5 Major Volatile Metabolites in Pear Juices

In previous studies, over 300 volatile metabolites have been identified in fresh pears and processed pear products.^43^ However, most of those compounds were detected in trace amounts, and only a fraction of key volatile metabolites (depending on their quantitative abundance and olfactory thresholds) were reported to play important roles in pear juices to provide pleasant fruity aroma.^43^ Thus, it is important to identify and quantify the major volatile metabolites in the studied pear juices. The key volatile metabolites were detected in the studied pear juices in the current study, including five esters, four alcohols, three aldehydes, and one volatile acid (Table 5). The total concentrations of quantified esters (sum contents of identified esters) were 70.5–217.8 μg/L in the studied pear juices. Among the studied pear cultivars, “Py1” (217.8 μg/L) contained the highest amounts of total quantified esters, followed by “Cla” (200.9 μg/L) and “Py12” (200.4 μg/L). In general, a high concentration of esters exerted strong ester notes; thus, the breeding selections “Py1” and “Py12” contained highest concentrations of esters among all the breeding selections. The dominant ester existed in juices of these three cultivars (“Py1”, “Cla”, and “Py12”) was detected as *n*-propyl acetate. Moreover, ethyl acetate was found to be the dominant ester in “Sto”, “Py8”, “Py10”, and “Con”. In addition, “Sto”, “Py3”, and “Py6” were found to have low total quantified ester contents of 70.5, 89.2, and 94.8 μg/L, respectively.

**Table 5:** Concentrations of Quantified Main Volatile Compounds in the Studied Pear Juices (μg/L)^a^

pear	ethyl acetate	methyl acetate	butyl acetate	*n*-propyl acetate	hexyl acetate	sum of quantified esters	propan-1-ol	ethanol	butan-1-ol	hexan-1-ol	sum of quantified alcohols	acetaldehyde	hexanal	(*E*)-2-hexenal	sum of quantified aldehydes	acetic acid
Py1	32.12	16.91	41.86	64.43	61.63	217.81	0.83	21.40	3.24	3.46	28.93	5.12	7.87	6.13	19.12	5.80
Py2	19.86	6.83	22.35	34.15	21.47	105.47	0.53	13.75	6.65	6.71	27.64	15.07	5.24	4.12	24.44	8.30
Py3	21.67	7.90	18.20	21.39	19.37	89.18	0.34	8.17	6.37	3.81	18.70	4.53	4.32	2.70	11.55	2.76
Py4	56.41	11.57	29.80	61.48	16.84	176.43	0.54	13.91	9.84	9.10	33.39	10.73	0.96	0.63	12.32	3.41
Py5	32.53	10.90	32.20	26.81	34.14	136.81	0.71	28.89	9.14	7.33	46.07	4.19	8.51	4.81	17.5	3.57
Py6	21.71	5.29	22.45	14.68	29.85	94.83	0.89	28.78	4.51	1.54	35.13	11.97	6.13	3.82	21.92	3.15
Py7	26.93	12.35	28.52	40.44	31.97	140.77	0.22	10.65	5.44	3.38	19.69	21.42	2.47	2.06	25.96	5.43
Py8	42.79	14.77	31.57	27.57	25.42	142.45	0.69	33.17	8.76	9.41	52.04	7.35	8.09	6.62	22.06	3.15
Py9	38.17	7.82	30.98	38.58	20.80	136.77	0.36	41.60	5.12	5.97	53.05	9.46	1.10	0.34	10.91	6.58
Py10	53.63	10.99	31.55	30.67	27.89	154.76	0.89	13.46	5.41	11.54	31.30	11.07	12.40	6.23	29.71	5.81
Py11	26.56	8.37	38.06	31.07	29.53	135.08	0.39	28.14	6.26	8.84	43.63	15.23	8.95	4.90	29.08	2.88
Py12	29.16	18.43	40.53	79.07	32.64	200.42	0.73	25.52	11.20	25.37	62.81	21.41	9.95	4.52	35.88	5.64
Py13	45.93	13.00	28.58	51.29	25.93	165.10	0.37	10.54	8.33	19.22	38.47	25.97	5.39	3.57	34.94	4.77
Sto	30.93	7.47	10.56	12.42	8.81	70.53	0.34	8.13	2.83	2.34	13.64	7.71	1.44	0.88	10.03	6.42
Kru	21.09	15.58	28.95	45.13	28.37	139.73	0.64	17.98	8.54	14.42	41.59	11.78	4.28	3.34	19.40	6.58
Con	65.84	13.48	21.04	50.67	16.24	168.36	0.64	22.64	13.40	12.75	49.43	21.60	10.8	5.76	38.11	7.19
Cla	56.73	12.08	46.04	60.51	24.38	200.87	0.69	4.81	8.52	18.33	32.35	18.23	12.7	6.29	37.22	5.40

a Results are presented as the average of triplicates. Abbreviations of pear cultivars refer to Table 1. The compound codes relate to the volatile compounds were followed as ethyl acetate (51), methyl acetate (52), butyl acetate (53), *n*-propyl acetate (54), hexyl acetate (55), propan-1-ol (56), ethanol (57), butan-1-ol (58), hexan-1-ol (59), acetaldehyde (60), hexanal (61), (*E*)-2-hexenal (62), and acetic acid (63). Complete information with standard deviation and significant differences are shown in Table S5***.***

Alcohols were detected as the second-dominant volatile groups in the studied pears (Table 5). The concentration of this group of compounds varied significantly among different pear cultivars. “Py12”contained the highest content of total quantified alcohols (sum of individual alcohols) at 62.8 μg/L, whereas “Sto” had a low content of total quantified alcohols at 13.6 μg/L. Ethanol was found in all studied juices, with contents ranging from 4.8 (“Cla”) to 41.6 μg/L (“Py9”). Butan-1-ol (3.2–13.4 μg/L) and hexan-1-ol (1.5–25.4 μg/L) showed relatively high concentrations in the studied pear juices, and their concentrations were mainly dependent on the pear cultivars. In addition, propan-1-ol showed similar low contents in the studied pear juices (0.2–0.9 μg/L).

For other volatile compounds, acetaldehyde was the most abundant aldehyde in the studied juices (Table 5). The concentration of acetaldehyde was mainly cultivar dependent, ranging from 4.2 μg/L in “Py5” to 26.0 μg/L in “Py13”. Apart from acetaldehyde, two C6 aldehydes, hexanal and (*E*)-2-hexenal, also showed relatively high amounts among the studied pear juices. The (*E*)-2-hexenal concentration varied dramatically from 0.3 μg/L in “Py9” to 6.6 μg/L in “Py8”. In addition, the concentration of acetic acid varied among juices, ranging from 2.8 (“Py3”) to 8.3 μg/L (“Py2”). All the volatile compounds were cultivar dependent in this study.

### 3.6 Association of Putative Pear Types with Chemical Profiles of Pear Juices

**Figure 1:**
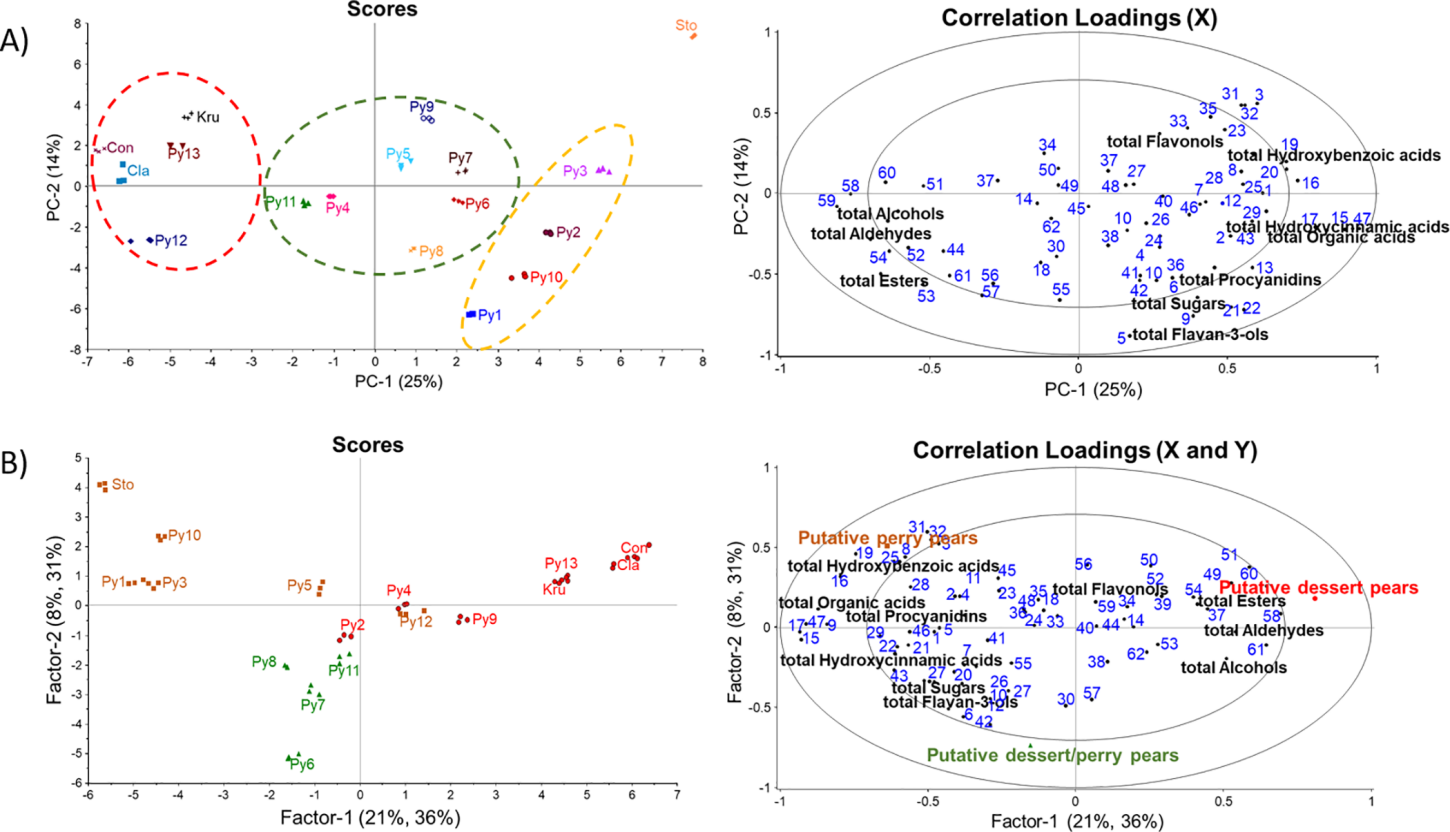
PCA (A) and PLS-DA (B) models of chemical compositions in pear juices (17 samples, 77 chemical compounds). In (A), pear cultivars are shown in different colors and symbols, whereas the putative dessert pears are indicated with red circles, putative perry pears with yellow rectangles, and putative dessert/perry pears with green triangles in (B). For sample and chemical compound codes, refer to Tables 1, 2, 4, and 5.

To assess the overall cultivar differences in the chemical compositions of pear juices, all the data (77 X-variables with 51 samples) regarding the phenolic compounds, major volatile compounds, sugars, and organic acids were analyzed using the PCA (Figure 1A) model. As shown in the PCA model, the first two principal components explained 39% of the total variance, with PC1 and PC2 accounting for 25 and 14%, respectively. “Sto” was clearly separated from the other pear cultivars and located on the positive side of PC1, with a strong correlation with hydroxybenzoic acids and hydroxycinnamic acids, primarily sinapic acid hexoside II (29), syringic acid hexoside I (3), quercetin hexoside deoxyhexoside I (31), quercetin hexoside deoxyhexoside II (32), and caffeic acid (16). Moreover, pear selections “Py1”, “Py2”, “Py3”, and “Py10” could be grouped together based on their similar chemical profiles, explained by the high amounts of total quantified organic acids, total quantified procyanidins, total quantified sugars, and total quantified flavan-3-ols, mainly as 4-*O*-caffeoylquinic acid (17), 5-*O*-caffeoylquinic acid (15), caffeoyl *N*-tryptophan (13), caffeoylhexose (30), coumaroylquinic acid isomer II (22), syringic acid hexoside II (9), procyanidin dimer B2 (6), (+)-catechin (5), and succinic acid (46). In contrast, cultivars “Con”, “Cla”, “Py13”, and “Py12” were located on the negative side of PC1 due to the higher amounts of major volatile compounds, primarily *n*-propyl acetate (54), butyl acetate (53), hexan-1-ol (59), butan-1-ol (58), and acetaldehyde (60). The pear selections “Py4”, “Py5”, “Py6”, “Py7”, “Py8”, and “Py11” were located in the middle part of the PCA plot. The PCA results also showed a varietal effect, which was in opposite directions between 5-*O*-caffeoylquinic acid (15), caffeic acid (16), 4-*O*-caffeoylquinic acid (17), and malic acid (47) on the one side (positive) and volatile compounds butyl acetate (53), *n*-propyl acetate (54), ethanol (57), and butan-1-ol (58) on the other side of the plot (negative). In addition, the Pearson correlation coefficient heatmap (Figure S2) revealed a significant bivariate connection between these compound variables in pear juices, which was supported by hierarchical co-clustering of the samples. The high contents of hydroxycinnamic acids in pear cultivars “Py1”, “Py2”, “Py3”, “Py10”, and “Sto” indicate high natural antioxidative and antimicrobial capacities, potentially protecting from natural harms by constituting a secondary reactive oxygen species (ROS) scavenging system in plants.^44,45^ Various studies have investigated the positive correlation between the phenolic concentrations and antioxidant activities for human nutrition, providing with anticancer, anti-inflammatory, antimicrobial, and antidiabetic activities.^46,47^ In contrast, pear breeding selections “Py12” and “Py13” together with the commercial cultivars “Con” and “Cla” were correlated closely with the aforementioned volatile compound variables, and thus the cultivars may be considered as putative dessert pears. Generally, dessert pears have more pleasant flavors due to the relatively high amounts of attractive volatile compound and less phenolic compounds. Therefore, understanding the biosynthesis of the potential flavor-active compounds and their interactions are required to more efficiently develop cultivars for different purposes.

The 17 studied different cultivars and breeding selections were classified into three groups (putative dessert pears, putative perry pears, and putative dessert/perry pears) according to their tentative use determined by breeders. Currently, “Sto”, “Py1”, “Py3”, “Py5”, “Py10”, and “Py12” are grouped as putative perry pears; “Py6”, “Py7”, “Py8”, and “Py11” are grouped as putative multiuse dessert/perry pears; and “Py2”, “Py4”, “Py9”, “Py13”, “Kru”, “Con”, and “Cla” are grouped as putative dessert pears. The differences among the putative dessert pear group, putative perry pear group, and putative dessert/perry pear group (*Y*-data, *n* = 3) in the chemical compositions (*X*-data, *n* = 77) were analyzed using PLS-DA (Figure 1B). In the PLS model with five validated factors (*R*^2^ = 0.9307, validated *R*^2^ = 0.8902), these three groups were separated well from each other. Overall, the putative perry pears were located on the negative side along Factor 1, with higher contents of total quantified hydroxybenzoic acids, total quantified hydroxycinnamic acids, and total quantified organic acids. In contrast, putative dessert pear group was located on the positive side along Factor 1. Interestingly, the full-sib pear selections of breeding program with the same parental cultivars (Py1-Py5) were divided into perry or dessert groups, result not fully unexpected by the complex inheritance and low or moderate heritability of these traits in pear.^26−28^ Moreover, the putative dessert/perry pear group was separated well from the other samples along Factor 2 and was located on the negative side of Factor 2. However, “Py12” (putative perry pears) was located close to the putative dessert pears in the PLS-DA model, and similar results were observed in the PCA model (Figure 1A). Moreover, “Py2” (putative dessert pears) was grouped together with the putative perry pears due to the higher contents of total quantified hydroxycinnamic acids, total quantified flavan-3-ols, total quantified procyanidins, total quantified sugars, and total quantified organic acids.

In conclusion, phenolic compounds, physiological characteristics, and other chemical compounds (sugars, organic acids, and major volatile metabolites) were investigated comprehensively in pear juices made from 17 pear cultivars, including 13 pear breeding selections, 2 test cultivars, and 2 commercial dessert pears. A total of 39 phenolic compounds were identified and quantified in the 17 studied pear cultivars. The genetic background effect on the phenolic profiles of pear juices is complex, and the chemical compositions of the breeding selections with the same parental cultivars varied dramatically from cultivar to cultivar. In general, the putative dessert group contained higher amounts of major volatile metabolites, primarily as *n*-propyl acetate, butyl acetate, hexan-1-ol, butan-1-ol, and acetaldehyde. The putative perry pear group correlated closely with 4-*O*-caffeoylquinic acid, 5-*O*-caffeoylquinic acid, coumaric acid derivative, caffeoylshikimic acid, coumaroylquinic acid isomer II, syringic acid hexoside II, procyanidin dimer B2, (+)-catechin, and succinic acid. However, as exceptions, juices made from “Py12”, putative perry type (determined by breeders) contained high volatile metabolites, whereas “Py2” (putative dessert pear) contained high phenolic compounds. The study provides a theoretical basis for product development to promote the utilization of local pear cultivars developed and grown in Finland. The potential of using the breeding selections in perry making deserves more investigation in the future.

## Supporting Information Available

(Figure S1) UHPLC-DAD chromatographs of phenolic compounds in pear juices; (Figure S2) Pearson heatmap of the selected chemical compounds; (Table S1) calibration information of volatile and phenolic compounds; (Table S2) physiochemical characteristics of the studied juice samples; (Table S3) quantified concentrations of individual phenolic compounds and major phenolic groups in pear juices (mg/L); (Table S4) quantified concentrations of sugars and organic acids in the studied pear juices (g/L); (Table S5) quantified concentrations of main volatile compounds in the studied pear juices (μg/L) (PDF)
