# Supplementary material for: Chemical Composition of Juices Made from Cultivars and Breeding Selections of European Pear (Pyrus communis L.)
Source: J Agric Food Chem. 2022 Apr 15;70(16):5137–50. doi: 10.1021/acs.jafc.2c00071 (PMC9052750; doi:10.1021/acs.jafc.2c00071)
Supplement: Supplementary file 1 — jf2c00071_si_001.pdf [file jf2c00071_si_001.pdf]

Characterization and quantification of phenolic compounds and other main metabolites in juices made from cultivars and breeding selections of European pear

Wenjia He[1], Oskar Laaksonen[1], Ye Tian[1], Tuuli Haikonen[2], Baoru Yang[1,*]

[1] Food Chemistry and Food Development, Department of Life Technologies, University of Turku, FI-20014 Turku, Finland

[2] Natural Resources Institute Finland (Luke), Production systems/Horticulture Technologies, Toivonlinnantie 518, FI-21500 Piikkiö, Finland

Corresponding author: Baoru Yang, baoru.yang@utu.fi, tel. +358 452737988

Figure S1. UHPLC-DAD chromatographs of phenolic compounds in pear juices.

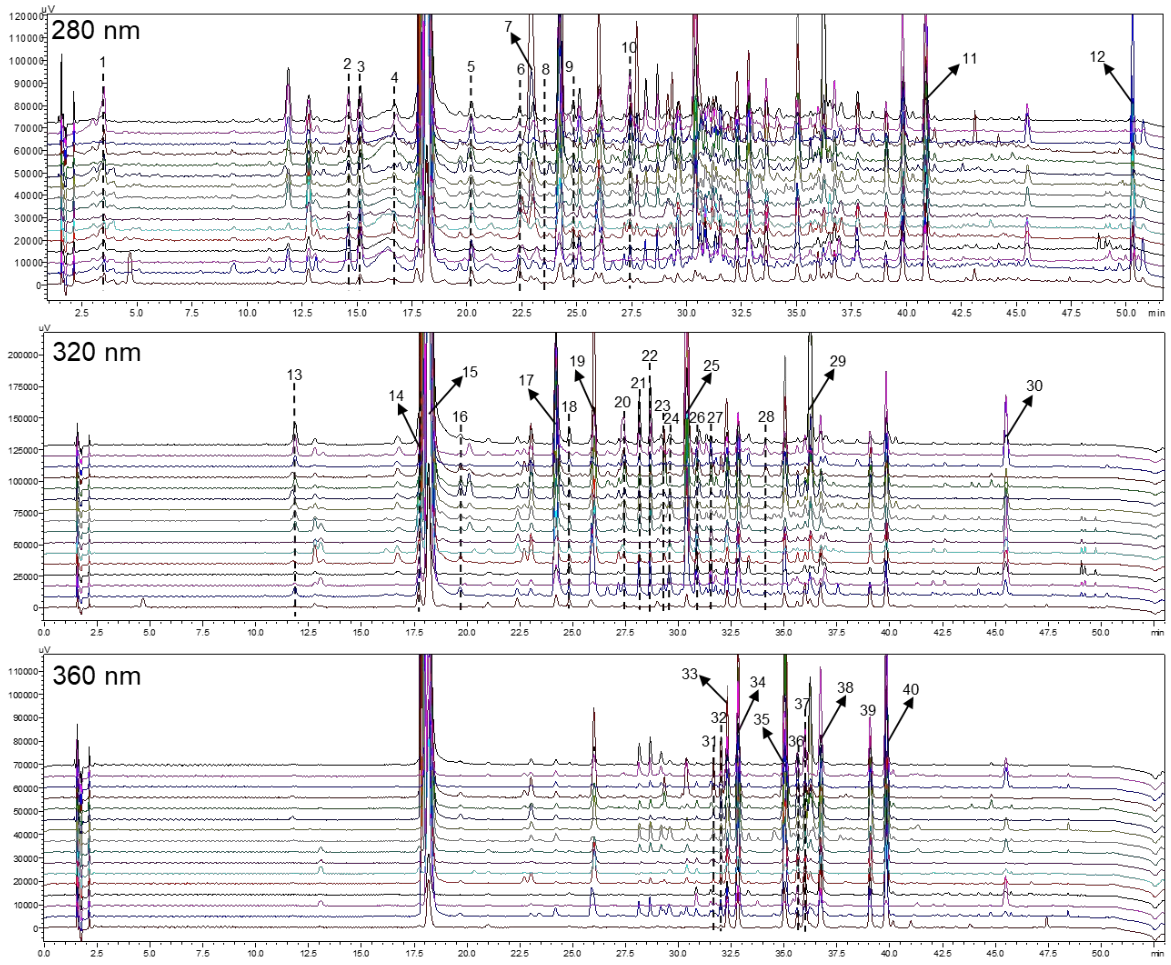

The chromatograms at 280 nm refer to hydroxybenzoic acids, flavan-3-ols, and procyanidins, the chromatograms at 320 nm refer to phenolic acids, and the chromatograms at 360 nm refer to flavonols. The peak numbers in the chromatograms refer to those in **Table 2**.

Figure S2. Pearson heatmap of the selected chemical compounds.

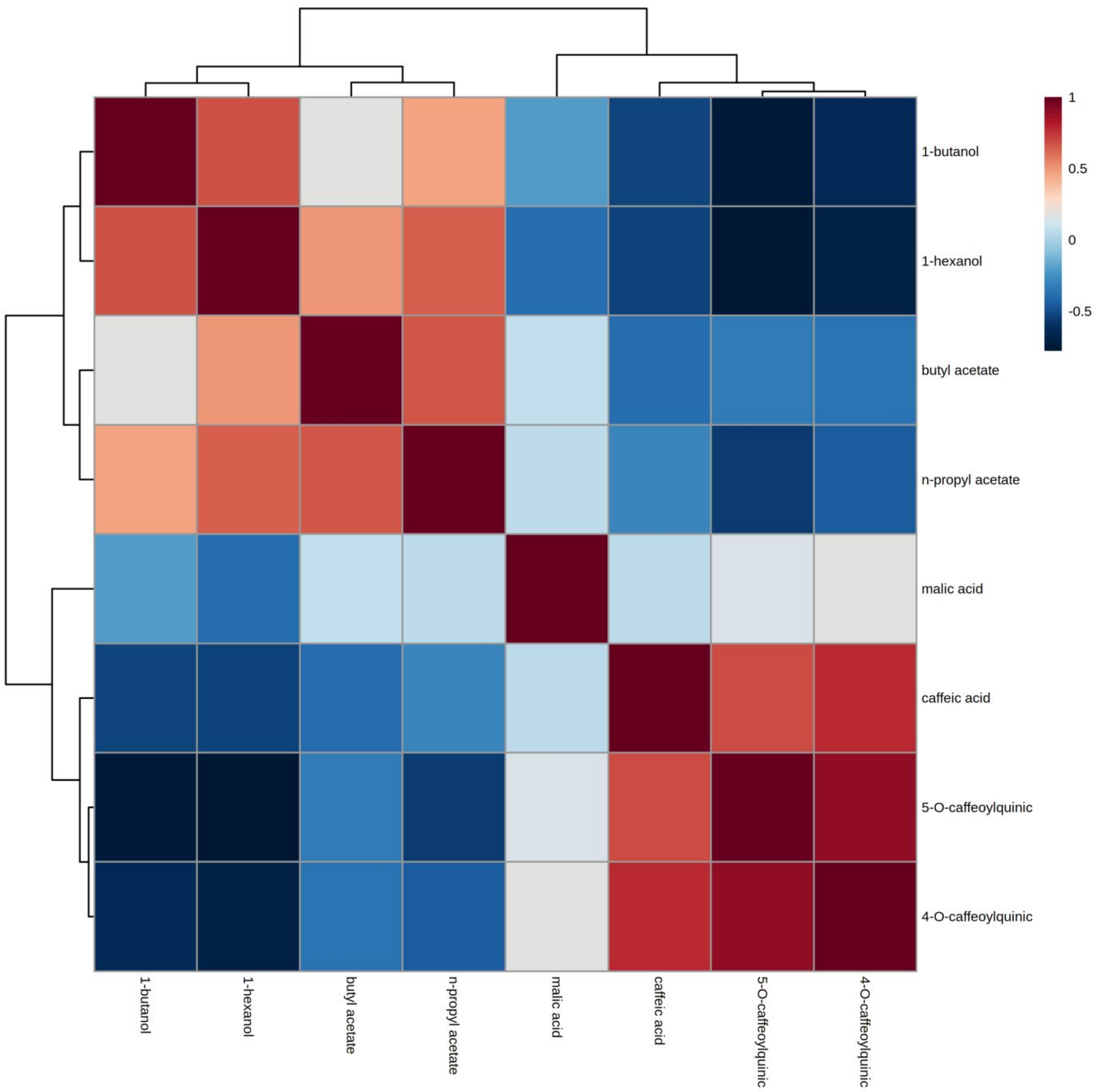

The selected chemical compounds are shown as rows and columns. Each square represents the Pearson correlation coefficient between two chemical compounds. The colour scheme from blue to red shows the normalized value from low to high.

Table S1. Calibration information of chemical compounds.

compounds	calibration curve	determination coefficient (R^2)	used for compound groups
acetaldehyde	y = 95548x - 25695	0.9995	aldehydes
ethyl acetate	y = 100429x - 25146	0.9992	esters
butan-1-ol	y = 144006x - 19876	0.9995	alcohols
acetic acid	y = 47598x - 1211.3	0.9991	acetic acid
5-*O*-caffeoylquinic acid	y = 53840050.42x -31439.07	0.9999	hydroxycinnamic acids
gallic acid 4-*O*-glucoside	y = 64254152.38x -6891.2	0.9999	hydroxybenzoic acids
(+)-catechin	y = 7032238.12x - 1213.24	0.9999	monomeric flavan-3-ols
PC dimer B2	y = 4416849.83x - 4225.15	0.9999	procyanidins
quercetin-3-*O*-glucoside	y = 10023284.12x + 1707.67	0.9999	flavonols
arbutin	y = 6598125.29x - 2596.36	0.9998	other phenolics

For the analysis of sugars and organic acids, myo-inositol and tartaric acid were used as internal standards for sugars and organic acids.

Table S2. Physicochemical characteristics of the studied pear cultivars

pear cultivars	pH	°Brix	juice yield (%)	tonality	color intensity
Py1	3.96±0.08c	15.40±0.35g	47.69±1.23bc	1.37±0.06a	6.81±0.35f
Py2	3.71±0.03b	11.85±0.20d	52.76±0.59d	1.86±0.12c	5.34±0.37d
Py3	3.35±0.12a	15.60±0.14g	42.95±0.69a	2.17±0.09d	0.27±0.01a
Py4	3.93±0.20c	11.45±0.20d	59.21±1.23e	1.48±0.14a	6.38±0.21f
Py5	3.48±0.02ab	11.80±0.30d	49.45±0.45c	1.98±0.15cd	2.35±0.27b
Py6	3.86±0.11bc	13.90±0.19f	56.53±0.32de	1.58±0.16ab	6.10±0.27ef
Py7	3.52±0.07ab	11.45±0.20d	74.93±0.68f	1.84±0.06c	2.39±0.22b
Py8	3.86±0.09bc	16.70±0.30h	46.06±0.53b	1.85±0.07c	4.17±0.23c
Py9	3.75±0.16b	15.20±0.15g	57.60±0.59de	1.66±0.03ab	5.55±0.24d
Py10	3.72±0.04b	11.10±0.10cd	54.81±0.82d	1.62±0.15ab	6.47±0.34f
Py11	3.85±0.07bc	12.40±0.25e	45.36±0.59b	1.49±0.09a	6.51±0.36f
Py12	3.93±0.11c	14.10±0.20f	49.03±1.05c	2.02±0.19cd	0.19±0.03a
Py13	4.24±0.02d	9.95±0.25b	56.83±0.71de	1.57±0.08ab	6.04±0.54e
Sto	3.31±0.05a	8.45±0.10a	83.97±0.84g	1.53±0.09a	6.67±0.13f
Kru	3.72±0.04b	10.90±0.15c	80.92±0.78g	1.62±0.16ab	6.47±0.21f
Con	4.55±0.10e	11.50±0.15d	58.07±1.03e	1.48±0.20a	4.36±0.21c
Cla	4.54±0.10e	12.70±0.20e	61.90±0.75e	1.41±0.17a	5.83±0.22de

Results are presented as the average of triplicates±standard deviation.

Significant differences among 17 pear juices are shown with lower case letters a-g (one way ANOVA with Tukey's post hoc test, $p < 0.05$).

Abbreviations of pear cultivars refer to **Table 1**.

Table S3. Concentrations of individual phenolic compounds in pear juices (mg/L).

pears	3	8	9	TBA	14	13	15	16	17	18
Py1	30.85±2.15e	0.97±0.13a	14.49±2.18bc	48.68±0.18c	ND	6.32±0.73g	195.41±10.04f	3.64±0.10d	26.25±3.36c	4.17±0.52e
Py2	28.82±3.14e	ND	12.16±0.18b	79.38±4.17e	2.96±0.13d	8.01±0.29h	184.31±14.24f	ND	22.95±2.13bc	ND
Py3	11.76±0.25c	ND	55.90±3.54e	75.53±5.69e	3.20±0.19d	2.85±0.32e	201.44±5.43fg	6.18±0.13f	36.54±4.50d	1.03±0.04c
Py4	19.98±1.15d	ND	11.36±0.35b	31.34±1.44b	4.06±0.09e	3.21±0.28e	79.76±5.84c	1.00±0.03a	24.36±3.23c	2.70±0.14e
Py5	17.12±0.13d	ND	30.42±5.67d	49.30±6.56c	3.96±0.07e	0.21±0.03a	155.63±11.26e	2.74±0.04b	19.09±4.12b	1.27±0.05c
Py6	13.53±0.14c	1.94±0.13b	4.70±0.15a	20.17±0.29a	ND	4.05±0.29f	158.66±6.57e	1.03±0.09a	20.42±2.32bc	0.88±0.10b
Py7	6.14±0.17b	ND	17.88±0.17c	29.49±0.20b	2.18±0.12b	3.91±0.16f	155.40±3.58e	3.05±0.15c	24.94±2.14c	0.86±0.08bc
Py8	35.50±4.27f	ND	12.94±0.42b	59.62±3.06d	2.62±0.03c	1.41±0.09c	149.61±3.79e	0.67±0.12a	18.47±1.48b	ND
Py9	8.76±0.51b	ND	15.20±1.53c	36.30±1.31b	5.90±0.18g	2.09±0.65d	117.74±1.36d	1.03±0.04a	16.01±1.14b	0.22±0.03a
Py10	27.37±2.23e	5.02±0.10c	16.81±3.16c	99.12±12.19f	5.47±0.14g	2.09±0.12d	200.68±10.77fg	3.04±0.06c	37.61±7.75d	1.00±0.05c
Py11	23.58±3.98de	ND	16.96±2.14c	40.54±0.85bc	8.48±1.54h	0.53±0.09b	161.13±9.98e	ND	17.46±2.38b	1.00±0.03c
Py12	23.80±5.17de	ND	11.30±0.70b	35.10±0.63b	4.52±0.12f	0.27±0.05a	51.05±0.90b	0.98±0.09a	7.29±0.21a	0.74±0.05b
Py13	16.92±0.10d	ND	6.10±0.68a	23.02±0.77a	ND	0.12±0.01a	37.05±0.21ab	ND	3.24±0.31a	2.10±0.10d
Sto	17.22±2.51d	5.76±0.11c	91.41±7.68f	127.22±12.61g	0.99±0.11a	1.33±0.18c	217.39±18.57g	5.09±0.08e	30.61±5.46cd	ND
Kru	7.99±0.66b	ND	11.90±0.59b	24.65±0.34a	3.89±0.10e	ND	26.52±0.74a	ND	4.39±0.09a	0.32±0.09a
Con	7.28±0.60b	ND	11.60±0.25b	18.88±0.74a	2.25±0.17b	ND	38.67±0.85ab	ND	2.90±0.08a	0.93±0.04bc
Cla	2.52±0.23a	ND	28.37±1.23d	30.90±1.43ab	3.19±0.20d	ND	36.88±4.36ab	ND	1.97±0.09a	1.02±0.02c

Table S3 *(continued)*

pears	19	20	21	22	23	24	25	26	27	28	29
Py1	7.67±0.92a	ND	12.37±2.30e	14.84±3.19e	0.99±0.04a	1.13±0.10b	14.51±3.71c	3.16±0.10d	1.33±0.05b	0.92±0.03a	17.14±3.40h
Py2	9.18±2.13a	11.39±2.58	8.81±1.22d	10.25±0.72e	0.93±0.04a	0.39±0.05a	27.67±4.60e	3.18±0.13d	ND	2.44±0.43b	ND
Py3	1.03±0.07a	2.57±0.11b	3.06±0.03b	3.03±0.03b	ND	ND	ND	2.56±0.12c	0.45±0.08a	ND	20.30±2.27i
Py4	4.03±0.07a	2.81±0.13b	ND	3.13±0.07b	0.82±0.03a	ND	8.51±0.49b	ND	ND	ND	1.15±0.11b
Py5	20.03±4.38b	3.92±0.04c	2.23±0.15a	3.38±0.53b	1.36±0.06b	ND	18.34±2.54cd	2.89±0.10c	ND	ND	0.38±0.03a
Py6	4.30±0.21a	5.33±0.29e	2.09±0.04a	6.25±0.87d	ND	ND	ND	5.36±0.49e	6.12±1.18g	ND	8.79±1.13g
Py7	8.76±1.16a	9.04±0.07g	5.12±0.09c	4.17±1.17c	6.57±1.48c	ND	18.87±2.54cd	5.40±0.39e	5.45±1.21f	ND	6.87±0.26f
Py8	12.49±3.54	2.89±0.18b	7.18±0.92d	6.98±0.93d	ND	ND	11.06±1.23c	0.65±0.05a	ND	0.91±0.05a	3.22±0.16d
Py9	2.14±0.15a	4.39±0.15d	2.03±0.03a	2.97±0.49b	0.98±0.07a	4.25±0.58c	ND	4.98±0.79e	0.76±0.06a	ND	5.12±0.63e
Py10	25.93±3.25b	3.14±0.12bc	9.05±0.68d	10.22±2.19e	0.46±0.06	4.11±0.19c	21.46±4.55d	5.54±0.63e	2.57±0.17d	ND	2.89±0.17d
Py11	8.75±0.19a	1.05±0.06a	ND	ND	ND	1.48±0.09b	8.80±0.84b	3.77±0.16d	ND	ND	2.28±0.17c
Py12	ND	ND	ND	ND	ND	ND	ND	4.15±0.14de	2.94±0.07e	ND	0.40±0.03a
Py13	ND	ND	ND	ND	ND	ND	ND	4.01±0.05de	2.04±0.07c	ND	0.34±0.02a
Sto	41.36±6.70c	7.56±0.42f	ND	1.12±0.18a	5.68±0.62c	ND	31.65±3.45e	2.53±0.19c	2.66±0.06d	2.26±0.15b	6.97±0.12f
Kru	7.44±0.45a	ND	ND	ND	ND	ND	7.61±0.39b	1.58±0.18b	2.09±0.03c	ND	ND
Con	3.10±0.14a	ND	ND	ND	ND	ND	1.48±0.44a	ND	ND	ND	ND
Cla	3.07±0.06a	ND	ND	ND	ND	ND	2.09±0.09a	ND	ND	ND	0.32±0.03a

Table S3 *(continued)*

pears	30	TCA	5	7	TFA	2	4	6	10	11
Py1	0.88±0.09b	304.41±18.01h	20.75±3.43c	11.61±0.96c	32.36±4.36d	2.38±0.28a	7.61±0.33ab	15.79±2.13d	12.49±2.39c	10.60±1.27ab
Py2	7.14±2.19e	291.60±16.74h	20.23±0.62c	12.47±2.50c	32.70±3.12d	38.41±4.28d	21.66±4.56d	20.03±0.24d	11.98±0.19bc	17.58±2.35b
Py3	ND	281.38±19.41h	12.49±2.19b	75.12±1.09g	87.61±3.28h	7.87±0.54b	9.00±0.45b	27.25±1.29f	2.01±0.14a	23.78±3.28c
Py4	1.00±0.04b	133.37±5.99c	14.77±0.65bc	12.84±0.12c	27.61±0.77c	ND	2.91±0.11a	17.93±1.05d	10.51±0.39bc	28.40±4.52d
Py5	2.16±0.14c	237.35±14.81f	10.79±0.56ab	21.27±5.26d	32.06±5.82d	1.76±0.19a	4.97±0.37ab	1.24±0.13a	1.87±0.10a	6.99±0.21a
Py6	8.37±1.34e	227.59±12.65ef	12.87±1.14b	14.12±1.27c	26.99±2.41c	ND	3.67±0.19a	25.47±3.03e	10.94±0.13bc	22.83±4.08c
Py7	0.41±0.10a	257.10±10.39g	11.50±0.32b	21.28±3.20d	32.78±3.52d	5.46±0.13ab	12.84±2.19bc	13.13±0.13cd	18.44±3.32c	7.74±0.13a
Py8	5.16±0.23d	221.90±3.56e	24.94±1.33d	27.86±0.10e	52.80±1.43f	11.18±0.81c	8.38±0.70b	10.72±0.31c	7.14±0.20b	16.01±0.29b
Py9	ND	168.51±11.17d	9.72±0.29ab	12.66±3.93c	22.38±4.22bc	12.34±1.31c	6.75±0.85ab	15.75±3.08d	11.77±1.34bc	13.75±0.41ab
Py10	1.08±0.05b	334.26±22.77i	32.41±3.41e	27.58±0.33e	59.99±3.74g	49.92±6.23e	28.34±4.60e	19.74±4.07d	15.76±1.13c	56.48±6.05e
Py11	ND	214.21±12.80e	16.43±0.34c	44.16±0.19f	60.59±0.63g	ND	10.51±3.28b	15.82±2.14d	2.20±0.11ab	31.46±3.51d
Py12	10.86±3.24f	82.93±6.96b	17.53±0.30c	9.81±0.17bc	27.34±0.47c	ND	15.04±1.08c	12.29±1.17cd	11.95±2.09bc	18.26±1.41b
Py13	1.11±0.09b	49.90±0.41a	10.66±0.32ab	10.27±0.04a	20.94±0.36b	ND	10.79±2.09b	6.83±0.05b	5.44±0.16b	9.54±0.49a
Sto	ND	355.86±25.22i	ND	40.46±2.44f	40.46±2.44e	12.84±2.13c	11.77±0.15bc	ND	ND	23.90±0.75c
Kru	0.97±0.07b	54.81±0.12a	7.20±0.73ab	4.65±0.46a	11.85±1.19a	4.76±0.12ab	5.53±0.40ab	2.51±0.18a	3.14±0.07ab	11.27±0.30ab
Con	0.90±0.18b	50.23±3.89a	5.82±0.30a	7.88±0.74a	13.70±1.04a	ND	8.68±0.35b	ND	ND	29.21±0.63d
Cla	ND	48.54±4.36a	8.39±0.19a	16.16±2.53c	24.55±2.72bc	ND	7.05±0.12ab	7.72±0.11b	ND	16.79±0.20b

Table S3 *(continued)*

pears	TPY	31	32	33	34	35	36	37	38	39	40
Py1	46.48±0.34bc	ND	ND	3.08±0.04a	4.66±0.12a	4.46±0.12ab	1.11±0.09b	0.85±0.06ab	2.72±0.03c	1.70±0.07b	5.30±0.16b
Py2	71.24±4.58e	ND	ND	9.81±2.28b	10.26±2.15b	9.00±1.05b	4.14±0.38e	3.20±0.37e	7.16±0.08g	4.28±0.09d	17.08±2.09f
Py3	62.04±5.91d	0.62±0.03b	2.17±0.13d	17.35±3.02c	7.79±0.17ab	15.31±2.61c	4.75±0.27e	1.05±0.05b	3.75±0.08d	2.48±0.06b	7.12±0.42c
Py4	59.75±3.61d	ND	ND	5.97±0.19ab	7.34±0.55ab	6.51±0.31ab	1.21±0.13c	1.01±0.03b	3.14±0.10c	0.99±0.03a	5.80±0.14bc
Py5	15.07±0.33a	ND	1.01±0.03a	8.22±0.64b	7.81±1.11ab	13.42±1.20bc	6.96±0.51f	2.03±0.03c	4.77±0.09e	2.26±0.06b	8.45±0.26d
Py6	62.91±2.11d	0.38±0.03a	ND	3.50±0.06a	5.90±0.18a	8.03±0.08b	0.31±0.05a	0.72±0.03a	4.16±0.11d	3.16±0.08c	8.84±0.17d
Py7	52.15±3.36cd	ND	ND	10.42±2.11b	7.23±0.07ab	15.66±0.25c	5.07±0.68e	2.16±0.04c	3.13±0.07c	2.10±0.04b	5.88±0.11bc
Py8	42.24±1.15bc	ND	ND	7.38±0.22b	6.50±0.03ab	11.61±0.39bc	2.19±0.06c	1.93±0.04c	4.14±0.06d	3.46±0.24c	4.90±0.06b
Py9	48.03±1.46c	0.77±0.06b	1.57±0.09c	28.28±5.37d	34.93±5.24d	43.77±6.45e	0.51±0.04a	ND	2.87±0.08c	9.06±0.56f	15.25±1.38f
Py10	120.32±10.58f	1.34±0.06c	2.28±0.11d	6.67±0.49ab	9.08±0.73b	8.03±0.36b	10.83±2.13g	0.63±0.03a	4.07±0.05d	4.42±0.17d	10.62±0.41d
Py11	60.00±5.41d	ND	ND	4.58±0.19ab	6.01±0.07a	2.17±0.13a	1.04±0.02b	ND	1.95±0.03b	2.14±0.15b	9.20±0.47d
Py12	57.53±6.58d	ND	ND	1.48±0.43a	5.44±0.17a	1.34±0.06a	ND	ND	1.00±0.03a	ND	ND
Py13	32.60±3.62b	ND	ND	3.49±0.21a	6.11±0.21a	8.64±0.45b	0.96±0.07b	2.54±0.10d	2.20±0.11b	2.00±0.05b	7.58±0.08c
Sto	35.68±0.80b	3.87±0.49d	5.78±0.49e	9.84±0.92b	14.87±2.17c	19.89±3.18d	0.65±0.06a	0.90±0.01b	0.70±0.03a	2.17±0.08b	5.07±0.08b
Kru	22.45±0.35a	0.65±0.03b	1.02±0.04a	7.12±1.13b	9.66±2.25b	8.44±0.60b	0.92±0.04b	1.02±0.04b	2.22±0.09b	0.88±0.10a	4.89±0.10b
Con	37.89±4.87b	ND	ND	6.87±0.21ab	9.86±1.17b	3.15±0.10a	3.11±0.05d	3.39±0.13e	3.65±0.06d	4.63±0.04d	2.19±0.13a
Cla	31.56±0.34b	ND	ND	6.87±0.12ab	10.98±0.98b	2.86±0.16a	3.24±0.04d	3.30±0.20e	5.74±0.12f	5.14±0.09e	13.09±0.10e

Table S3 *(continued)*

pears	TFO	12	1	TPA
Py1	23.87±4.41b	24.59±0.42d	10.68±2.19b	552.38±40.05f
Py2	64.93±7.51e	59.60±3.65g	29.50±0.48d	688.88±55.64g
Py3	62.38±7.06e	30.56±0.52e	19.78±0.41c	658.32±35.69g
Py4	31.96±4.52bc	26.09±3.57d	9.97±0.42b	335.38±10.95c
Py5	54.94±6.56d	36.73±2.44ef	16.30±4.09c	443.81±31.23de
Py6	35.00±4.39b	70.93±5.63h	6.90±0.07a	486.56±38.44e
Py7	51.65±6.48d	28.31±1.57d	11.82±0.69b	494.49±23.53e
Py8	42.12±0.16c	60.28±4.46g	25.36±5.52d	496.12±24.92e
Py9	137.02±15.31f	38.60±1.10f	5.35±0.28a	469.52±32.88e
Py10	57.98±8.98d	18.58±0.26c	9.08±0.18b	714.55±45.26g
Py11	27.09±3.41b	23.36±0.49d	5.65±0.35a	406.93±25.89d
Py12	9.26±0.56a	14.58±0.20b	4.38±0.28a	234.76±17.36b
Py13	33.52±0.15b	32.33±3.07e	9.64±0.25b	198.00±9.95a
Sto	63.74±7.32e	34.12±2.83ef	18.00±2.62c	654.28±32.57g
Kru	36.79±2.34bc	23.67±1.84d	3.33±0.32a	177.54±12.15a
Con	36.84±4.22b	8.91±0.11a	6.42±0.14a	172.87±9.79a
Cla	51.22±7.08d	17.34±0.58c	5.57±0.23a	209.68±14.71ab

Results are presented as the average of triplicates ± standard deviation.

The concentrations of hydroxycinnamic acids, hydroxybenzoic acids, monomeric flavan-3-ols, procyanidins, flavonols, and arbutin are presented as equivalents of 5-O-caffeoylquinic acid, (+)-catechin, procyanidin B2, quercetin-3-O-glucoside, and arbutin, respectively.

TBA: total quantified hydroxybenzoic acids, TCA: total quantified hydroxycinnamic acids, TFA: total quantified flavan-3-ols, TPY: total quantified procyanidins, TFO: total flavonols, TPA: total quantified phenolic compounds.

Significant differences among 17 pear juices are shown with lower case letters a-i (one way ANOVA with Tukey's post hoc test, $p<0.05$). Abbreviations of pear cultivars and phenolic compounds refer to **Table 1** and **Table 2**, respectively.

Table S4. Concentrations of sugars and organic acids in the studied pear juices (g/L).

pear	glucose	sucrose	fructose	sorbitol	xylose	total	succinic acid	malic acid	quinic acid	citric acid	ascorbic acid	total organic acids
Py1	30.53±0.85i	21.57±2.98ef	69.02±3.07f	20.08±1.00f	0.99±0.10cd	142.18±7.26f	0.54±0.03c	7.83±0.68h	1.32±0.12c	0.24±0.03c	0.34±0.03bc	10.26±0.26g
Py2	24.81±0.84h	16.10±0.75d	61.88±1.45e	9.21±0.17b	0.51±0.09b	112.51±1.44c	0.23±0.03a	6.11±0.41f	1.41±0.04c	0.13±0.04b	0.33±0.03bc	8.21±0.12ef
Py3	24.27±1.02h	17.74±0.70d	84.37±1.31h	14.93±0.34d	0.89±0.02c	142.19±1.33f	0.49±0.04b	8.61±0.21i	1.84±0.08e	0.12±0.01b	0.25±0.01ab	11.31±0.26h
Py4	20.75±0.96g	30.23±1.20hi	69.12±0.67f	14.41±0.74d	0.86±0.08c	135.37±1.15e	0.40±0.04b	4.42±0.10d	1.55±0.01cd	0.62±0.03d	0.46±0.06d	7.45±0.14de
Py5	14.69±0.13d	11.40±0.02b	54.56±0.37bc	6.37±0.08a	1.44±0.01e	88.46±0.36a	0.37±0.01b	5.88±0.04f	1.12±0.03b	0.19±0.01bc	0.29±0.00b	7.85±0.06e
Py6	15.28±1.06de	28.23±0.76h	68.84±1.47f	13.26±0.29d	0.72±0.08bc	126.34±2.98d	0.44±0.02b	4.75±0.11d	1.34±0.02c	0.13±0.01b	0.31±0.02b	6.97±0.08d
Py7	13.57±0.58cd	24.34±0.14g	54.21±0.63c	13.69±0.12c	0.44±0.04b	106.25±0.96bc	0.43±0.05b	4.73±0.17d	0.75±0.13a	0.15±0.05b	0.27±0.04ab	6.33±0.32cd
Py8	17.06±0.58ef	32.35±0.84i	83.27±0.57h	18.81±0.52e	0.53±0.08b	152.56±0.64g	0.39±0.02b	6.87±0.03g	1.11±0.10b	0.15±0.04b	0.31±0.02b	8.84±0.13f
Py9	10.15±0.70b	20.42±0.28e	62.61±1.23e	13.19±0.07d	0.43±0.07b	106.80±1.58bc	0.38±0.02b	4.02±0.03cd	1.46±0.12c	0.14±0.00b	0.22±0.00a	6.21±0.15cd
Py10	8.80±0.16a	22.51±0.16f	52.72±0.07b	13.56±0.16d	1.66±0.02f	99.25±0.25b	0.37±0.02b	5.11±0.09e	1.17±0.13b	0.20±0.00bc	0.43±0.07cd	7.28±0.23de
Py11	14.14±0.70d	19.17±0.74e	59.59±0.47d	19.19±0.98ef	1.85±0.08f	113.93±1.94c	0.32±0.01ab	3.53±0.09c	1.26±0.07bc	1.46±0.16f	0.41±0.08cd	6.97±0.37d
Py12	16.13±0.66e	28.82±1.02h	62.54±1.66e	24.52±1.00g	0.92±0.11cd	132.93±0.39e	0.33±0.01ab	2.95±0.09bc	1.38±0.06c	1.46±0.11f	0.39±0.13c	6.51±0.18d
Py13	10.76±0.39b	6.33±0.34a	52.25±0.44b	13.27±1.22d	1.05±0.02d	83.65±0.62a	0.39±0.02b	2.50±0.16b	1.72±0.04d	0.27±0.08c	0.37±0.07c	5.26±0.15c
Sto	7.38±0.59c	8.63±0.52c	42.10±0.16f	8.17±0.19d	1.16±0.10d	67.44±1.13bc	0.43±0.03b	7.22±0.15g	1.36±0.06c	1.14±0.07e	0.29±0.01b	10.44±0.13g
Kru	10.95±0.14b	10.33±0.05b	47.47±0.35a	11.42±0.09c	0.72±0.05bc	80.89±0.48a	0.38±0.01b	1.83±0.04a	1.00±0.02ab	0.03±0.01a	0.40±0.05cd	3.61±0.12a
Con	15.42±0.14d	11.14±0.10b	52.73±0.23b	17.93±0.11e	1.29±0.07d	98.50±0.42b	0.27±0.02a	2.73±0.06b	1.16±0.04b	0.08±0.02ab	0.49±0.12cd	4.73±0.08b
Cla	18.01±0.66f	13.72±0.23c	47.63±3.58a	23.09±0.92g	0.26±0.06a	102.71±4.77b	0.33±0.02ab	3.16±0.05bc	1.13±0.08b	0.20±0.01bc	0.70±0.02d	5.51±0.13c

Results are presented as the average of triplicates ± standard deviation. Significant differences among 17 pear juices are shown with lower case letters a-i (one way ANOVA with Tukey's post hoc test, $p < 0.05$). Abbreviations of pear cultivars refer to Table 1.

Table S5. Concentrations of main volatile compounds in the studied pear juices (µg/L).

pear	ethyl acetate	methyl acetate	butyl acetate	*n*-propyl acetate	hexyl acetate	sum of esters	propan-1-ol	ethanol	butan-1-ol	hexan-1-ol	sum of alcohols	acetaldehyde	hexanal	(*E*)-2-hexenal	sum of aldehydes	acetic acid
Py 1	32.12±1.11c	16.91±0.28f	41.86±4.19d	64.43±0.61f	61.63±6.48g	217.81±9.56i	0.83±0.03f	21.40±0.90f	3.24±0.33a	3.46±0.44a	28.93±1.30c	5.12±0.22a	7.87±0.22f	6.13±0.17ef	19.12±0.55bc	5.80±0.19c
Py 2	19.86±3.26a	6.83±0.47ab	22.35±2.89b	34.15±4.59d	21.47±2.53c	105.47±8.05c	0.53±0.04c	13.75±0.60d	6.65±0.18c	6.71±0.29b	27.64±0.58c	15.07±0.06e	5.24±0.32d	4.12±0.10c	24.44±0.17d	8.30±0.35f
Py 3	21.67±1.67a	7.90±0.44b	18.20±0.55b	21.39±1.08b	19.37±2.43bc	89.18±5.46b	0.34±0.02b	8.17±0.08b	6.37±0.13c	3.81±0.50a	18.70±0.08b	4.53±0.17a	4.32±0.24c	2.70±0.58b	11.55±0.77a	2.76±0.11a
Py 4	56.41±5.88f	11.57±1.45c	29.80±3.42c	61.48±5.73f	16.84±1.46b	176.43±9.25g	0.54±0.02c	13.91±0.63d	9.84±0.68d	9.10±0.06c	33.39±0.88d	10.73±0.65cd	0.96±0.04a	0.63±0.07a	12.32±0.66a	3.41±0.36a
Py 5	32.53±0.67c	10.90±0.22c	32.20±4.42c	26.81±3.57c	34.14±4.14f	136.81±6.85d	0.71±0.03e	28.89±0.66h	9.14±0.14d	7.33±0.04bc	46.07±0.50g	4.19±0.11a	8.51±0.48f	4.81±0.17d	17.50±0.65b	3.57±0.15a
Py 6	21.71±2.37a	5.29±1.46a	22.45±4.82b	14.68±0.41a	29.85±3.26e	94.83±5.72b	0.89±0.03f	28.78±3.65h	4.51±0.19ab	1.54±0.14a	35.13±2.47d	11.97±0.51d	6.13±0.22e	3.82±0.15c	21.92±0.77c	3.15±0.14a
Py 7	26.93±2.48b	12.35±0.28cd	28.52±3.48c	40.44±5.39d	31.97±3.66e	140.77±8.68d	0.22±0.01a	10.65±0.11c	5.44±0.15b	3.38±0.50a	19.69±0.30b	21.42±0.58g	2.47±0.17b	2.06±0.05b	25.96±0.63d	5.43±0.17c
Py 8	42.79±4.59e	14.77±0.67e	31.57±2.78c	27.57±4.35c	25.42±3.76d	142.45±6.23d	0.69±0.02de	33.17±3.60i	8.76±0.16d	9.41±0.37c	52.04±3.28i	7.35±0.06b	8.09±0.09f	6.62±0.28f	22.06±0.36c	3.15±0.13a
Py 9	38.17±4.09d	7.82±0.17b	30.98±2.30c	38.58±3.42d	20.80±2.38c	136.77±5.27d	0.36±0.03b	41.60±0.68j	5.12±0.25b	5.97±0.17b	53.05±0.71i	9.46±0.44c	1.10±0.09a	0.34±0.05a	10.91±0.55a	6.58±0.24d
Py 10	53.63±4.82f	10.99±0.52c	31.55±3.59c	30.67±1.46cd	27.89±4.25de	154.76±9.24e	0.89±0.02f	13.46±0.21d	5.41±0.37b	11.54±1.36d	31.30±2.90d	11.07±0.52d	12.41±0.36h	6.23±0.12ef	29.71±0.31e	5.81±0.15c
Py 11	26.56±2.50b	8.37±0.28b	38.06±4.00d	31.07±2.01cd	29.53±3.49e	135.08±7.68d	0.39±0.01b	28.14±0.22h	6.26±0.07c	8.84±0.14c	43.63±0.09f	15.23±0.10e	8.95±0.17fg	4.90±0.11d	29.08±0.16e	2.88±0.09a
Py 12	29.16±0.92c	18.43±0.90g	40.53±1.50d	79.07±6.52g	32.64±2.68ef	200.42±9.25h	0.73±0.04e	25.52±1.51g	11.20±2.88e	25.37±3.49g	62.81±6.23i	21.41±1.14g	9.95±0.12g	4.52±0.17cd	35.88±1.12f	5.64±0.09c
Py 13	45.93±2.69e	13.00±0.13d	28.58±3.51c	51.29±4.01e	25.93±0.11d	165.10±8.06f	0.37±0.02b	10.54±0.58c	8.33±0.29d	19.22±0.27f	38.47±0.19e	25.97±0.16h	5.39±0.28d	3.57±0.15bc	34.94±0.25f	4.77±0.10b
Sto	30.93±3.67c	7.47±0.23b	10.56±1.73a	12.42±1.36a	8.81±0.50a	70.53±5.16a	0.34±0.03b	8.13±1.57b	2.83±0.14a	2.34±0.09a	13.64±0.66a	7.71±0.33b	1.44±0.19ab	0.88±0.08a	10.03±0.40a	6.42±0.06d
Kru	21.09±3.86a	15.58±2.67ef	28.95±4.60c	45.13±6.65de	28.37±3.52e	139.73±7.88d	0.64±0.03d	17.98±0.69e	8.54±0.45d	14.42±0.07e	41.59±1.07f	11.78±0.37d	4.28±0.23c	3.34±0.11bc	19.40±0.56bc	6.58±0.27d
Con	65.84±4.71g	13.48±0.21d	21.04±1.09b	50.67±5.50e	16.24±0.84b	168.36±3.53f	0.64±0.01d	22.64±1.38f	13.40±0.63f	12.75±0.42d	49.43±4.02h	21.60±0.90g	10.75±0.07g	5.76±0.11e	38.11±0.82g	7.19±0.08e
Cla	56.73±2.01f	12.08±0.23cd	46.04±3.75e	60.51±3.45f	24.38±2.52d	200.87±6.93h	0.69±0.03de	4.81±0.21a	8.52±0.55d	18.33±0.52f	32.35±5.14d	18.23±0.42f	12.70±0.39h	6.29±0.09ef	37.22±0.78g	5.40±0.28c

Results are presented as the average of triplicates ± standard deviation. Significant differences among 17 pear juices are shown with lower case letters a-j (one way ANOVA with Tukey's post hoc test, $p<0.05$). Abbreviations of pear cultivars refer to **Table 1**.
